# A machine learning approach to predict metabolic pathway dynamics from time-series multiomics data

**DOI:** 10.1038/s41540-018-0054-3

**Published:** 2018-05-29

**Authors:** Zak Costello, Hector Garcia Martin

**Affiliations:** 1 Biological Systems and Engineering Division, Lawrence Berkeley National Laboratory, Berkeley, CA USA; 2 DOE Agile Biofoundry, Emeryville, CA USA; 3 DOE Joint BioEnergy Institute, Emeryville, CA USA; 4 BCAM, Basque Center for Applied Mathematics, Bilbao, Spain

**Keywords:** Synthetic biology, Metabolic engineering, Time series, Dynamical systems

## Abstract

New synthetic biology capabilities hold the promise of dramatically improving our ability to engineer biological systems. However, a fundamental hurdle in realizing this potential is our inability to accurately predict biological behavior after modifying the corresponding genotype. Kinetic models have traditionally been used to predict pathway dynamics in bioengineered systems, but they take significant time to develop, and rely heavily on domain expertise. Here, we show that the combination of machine learning and abundant multiomics data (proteomics and metabolomics) can be used to effectively predict pathway dynamics in an automated fashion. The new method outperforms a classical kinetic model, and produces qualitative and quantitative predictions that can be used to productively guide bioengineering efforts. This method systematically leverages arbitrary amounts of new data to improve predictions, and does not assume any particular interactions, but rather implicitly chooses the most predictive ones.

## Using artificial intelligence to predict biological dynamics

New synthetic biology capabilities (e.g. CRISPR) dramatically improve our ability to engineer biological systems for the benefit of society (biofuels, medical drugs). However, this effort is hampered because we cannot reliably predict the outcome of our bioengineering efforts. Mathematical kinetic models have been traditionally used to predict pathway dynamics, but they take a long time to develop and require significant biological expertize. Here, we substitute traditional kinetic models with a machine learning approach that is able to learn pathway dynamics straight from data examples. This new approach can be systematically applied to any product, pathway or host and significantly speeds up bioengineering.

## Introduction

Biology has been transformed in the second half of the twentieth century from a descriptive science to a design science. This transformation has been produced by a combination of the discovery of DNA as the repository of genetic information,^1^ and of recombinant DNA as an effective way to alter this instruction set.^2^ The subsequent advent of genetic engineering and synthetic biology as effective tools to engineer biological cells has produced numerous beneficial applications ranging from the production of renewable biofuels and other bioproducts^3–6^ to applications in human health,^7–9^ creating the expectation of an industrialized biology affecting almost every facet of human activity.^10^

However, effective design of biological systems is precluded by our inability to predict their behavior. We can engineer changes faster than ever, enabled by DNA synthesis productivity that improves as fast as Moore’s law,^11^ and new tools like CRISPR-enabled genetic editing, which have revolutionized our ability to modify the DNA in vivo.^12^ In general, we can make the DNA changes we intend (in model systems), but the end result on cell behavior is usually unpredictable.^13^ At the same time, there is an exponentially increasing amount of functional genomics data available to the experimenter in order to phenotype the resulting bioengineered organism: transcriptomics data volume has a doubling rate of 7 months,^14^ and high-throughput workflows for proteomics^15^ and metabolomics^16^ are becoming increasingly available. Furthermore, the miniaturization of these techniques and the progressive automation of laboratory work through microfluidics chips promises a future where data analysis will be the bottleneck in biological research.^17^ Unfortunately, the availability of all this data does not translate into better predictive capabilities for biological systems: converting these data into actionable insights to achieve a given goal (e.g., higher bioproduct yields) is far from trivial or routine.

Mathematical modeling provides a systematic manner to leverage these data to predict the behavior of engineered systems. Hence, increasingly, computational biology is focusing on large-scale modeling of dynamical systems predicting phenotype from genotype.^18,19^ However, computational biology is still nascent and not able to provide the high accuracy predictions that we are accustomed to seeing in other engineering fields.^20^ Arguably, the most widely used and successful modeling technique in metabolic engineering involves analysis of internal metabolic fluxes (i.e., reaction rates) through stoichiometric models of metabolism. Metabolic flux values are constrained by stochiometry, thermodynamic and evolutionary assumptions,^21,22^ or experimental data (e.g.,^13^C labeling experimental data^23–25^), and used to suggest genetic interventions that bring cell metabolism closer to the desired phenotype. While this approach has provided significant successes,^26–29^ it has also shown its limitations^30^ due to its simplicity. Stochiometric models are limited for bioengineering purposes because they ignore enzyme kinetics and cannot accurately capture dynamic metabolic responses, nor offer a straightforward way to leverage ever more abundant proteomics and metabolomics data for increased accuracy.

**Fig. 1:**
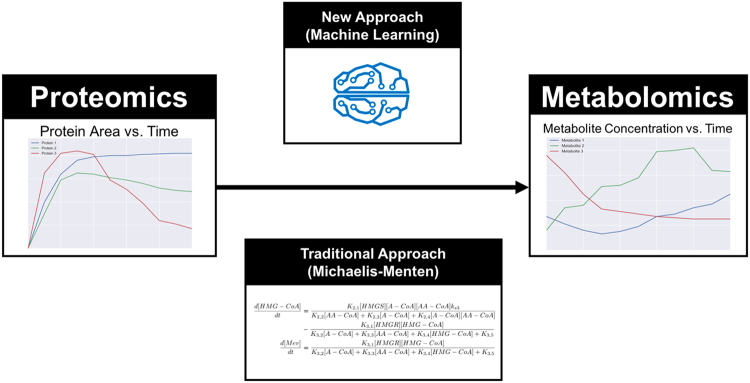
An alternative to traditional kinetic modeling by using machine learning. Our goal is to use time series proteomics data to predict time-series metabolomics data (Fig. 2). The traditional approach involves using ordinary differential equations where the change in metabolites over time is given by Michaelis–Menten kinetics (Figs. 3 and 4). The alternative approach proposed here uses time series of proteomics and metabolomics data to feed machine learning algorithms in order to predict pathway dynamics (Eq. (1) and Supplementary Fig. S1). While the machine learning approach necessitates more data, it can be automatically applied to any pathway or host, leverages systematically new data sets to improve accuracy, and captures dynamic relationships which are unknown by the literature or have a different dynamic form than Michaelis–Menten kinetics

Kinetic models explicitly take into account enzyme kinetics and are able to predict metabolite concentrations as a function of time from protein concentrations.^31^ This type of prediction is useful to metabolic engineers in order to design pathways that have the desired titers, rates, and yields. Kinetic models rest on an explicit functional relationship connecting the rate of change of a metabolite and the proteins and metabolites involved in the reaction (see Fig. 1): Michaelis–Menten kinetics^32,33^ is the most common choice, but the fact is that the true mechanistic kinetic rate law for each specific reaction is unknown for most enzymes^34^ (alternatives include generalized mass action,^35^ lin-log kinetics,^36,37^ or power-law models^38^). However, there is a lack of reliable data for the enzyme activity and substrate affinity parameters used in these models: in-vitro characterization may not be extrapolatable to in vivo conditions, and the effect of activators and inhibitors are typically unknown. Approaches such as ensemble modeling^39–43^ tackle the parsity of known kinetic constants by producing an ensemble of models displaying different combinations of randomly chosen kinetic parameters and selecting only those models that match known experimental data, or by optimizing the selection of these parameters through genetic algorithms.^44,45^ In a similar fashion, ORACLE^46,47^ produces populations of models which are consistent with reaction stochiometry, thermodynamics, and available concentration and fluxomic data. By design, these approaches are able to match measured final production levels and flux data, and the predictions have been shown to improve as the model approaches genome-scale coverage.^45^ However, a significant problem remains in that essential mechanisms are only sparsely known: allosteric regulation, for example, is known to be critical in order to determine fluxes,^48,49^ and yet a comprehensive map of this regulatory mechanism is unavailable. Post-translational modifications of proteins are also known to markedly affect catalytic activity,^50^ but are still largely unmapped. Pathway channeling, too, significantly affects catalytic rates but the degree to which this phenomenon occurs in metabolism has only begun to be explored.^51–53^ These and other gaps in our knowledge of mechanisms will require significant time and effort to be cleared. Given the urgent need of predictive capabilities by the emerging biotech industry, it may be useful to consider a different approach while this knowledge is gathered.

**Fig. 2:**
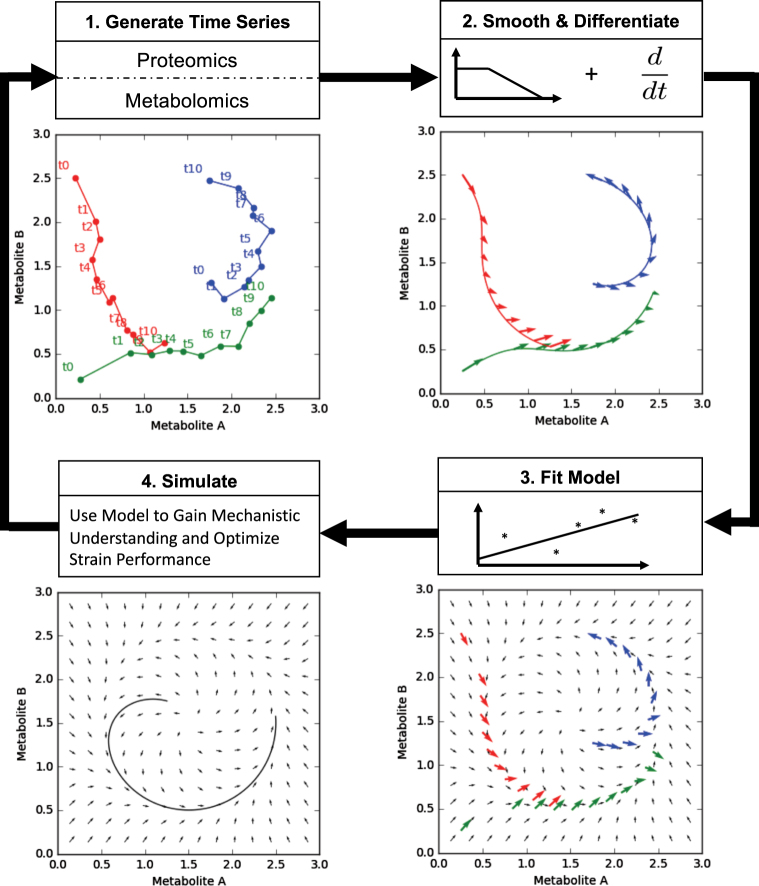
Cycle for learning metabolic system dynamics from time-series proteomics and metabolomics data. (1) Experimentally, time-series proteomics and metabolomics data are acquired for several strains of interest (represented by different colors). These data are represented in a metabolomics phase space, with an axis corresponding to each measured metabolite. (2) The time-series data traces are smoothed and differentiated (Supplementary Fig. S2). The derivatives provide the training data to derive the relationship between metabolomics and proteomics data and the metabolite change (Supplementary Fig. S1, Eq. (1)). (3) The state derivative pairs are fed into a supervised machine learning algorithm. The machine learning algorithm learns and generalizes the system dynamics from the examples provided by each strain. (4) The model can then be used to simulate virtual strains and explore the metabolic space looking for mechanistic insight or commercially valuable designs. This process can then be repeated using the model to create new strains, which will further improve the accuracy of the dynamic model in the next round

Here, we propose an alternative to traditional kinetic modeling involving a machine learning approach (Figs. 1 and 2), in which the function that determines the rate of change for each metabolite from protein and metabolite concentrations is directly learned from training data (Eq. (1) and Supplementary Fig. S1), without presuming any specific relationship. Machine learning has shown remarkable success in well bounded problems where a mechanistic model is impossible or difficult to develop: e.g., artificial vision for driverless cars,^54^ automated playing of the Go game,^55^ automated language translation^56^, or private trait prediction from digital records of human behavior^57^ with direct impact on national elections.^58^ In biology, these methods have recently been successfully applied to challenging problems such as predicting DNA and RNA protein-binding sequences,^59^ skin cancer diagnosis,^60^ single-nucleotide polymorphism (SNP) and small indel variant calling,^61^ and tumor detection in breast histopathology.^62^

**Fig. 3:**
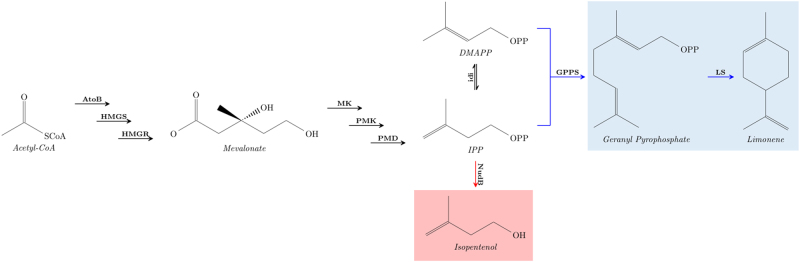
The new method was tested on the limonene and isopentenol metabolic pathways. The limonene (blue) and isopentenol (red) producing pathways are variants of the mevalonate pathway. Time-series proteomics and metabolomics data are used to learn the dynamics of both the isopentenol and limonene producing strains. Additionally, a kinetic model is created and compared to the machine learning approach for the more complex limonene production pathway (Fig. 4). This pathway model is also used to generate simulated data to further evaluate the scaling properties of the proposed machine learning approach. See the original data set^66^ for enzyme and metabolite acronyms

This alternative, machine-learning based, approach provides a faster development of predictive pathway dynamics models since all required knowledge (regulation, host effects… etc) is inferred from experimental data, instead of arduously gathered and introduced by domain experts (see Supplementary Material for an example). In this way, the method provides a general approach, valid even if the host is poorly understood and there is little information on the heterologous pathway, and provides a systematic way to increase prediction accuracy as more data is added. This method obtains better predictions than the traditional Michaelis–Menten approach for the limonene and isopentenol producing pathways studied here (Fig. 3) using only two times series (strains), and is shown to significantly improve its prediction performance as more time series are added. The new method is accurate enough to drive bioengineering efforts: we show it is able to predict the relative production ranking for several designs, given enough data. This approach is a specific solution to the more general type of problem of determining dynamics from observed data (system identification),^63–65^ a problem generally recognized as hard. We believe this approach is scalable to genome-scale models, or generally applicable to other types of data (e.g., transcriptomics) or dynamic systems (e.g., microbiome dynamics).

### Mathematical problem formulation

Here, we describe the problem and its solution in succinct mathematical terms. Let us assume we are given *q* sets of time series metabolite ${\tilde{\bf m}}^i[t] \in {\Bbb R}^n$ (Supplementary Fig. S2) and protein ${\tilde{\bf p}}^i[t] \in {\Bbb R}^\ell$ observations at times ${\bf{T}} = \left[ {t_1,t_2, \ldots ,t_s} \right] \in {\Bbb R}_ + ^s$. The superscript *i* ∈ {1, …, *q*} indicates the time-series index (strain), and ${\tilde{\bf m}}[t]$ = $\left[ {\tilde m_1[t], \ldots ,\tilde m_n[t]} \right]^T$ and ${\tilde{\bf p}}[t]$ = $[\tilde p_1[t], \ldots ,\tilde p_\ell [t]]^T$ are vectors of measurements at time *t* containing concentrations for the *n* metabolites and $\ell$ proteins considered in the model. We require the number of observation time points to be dense enough to capture the dynamic behavior of the system.

We also assume that the underlying continuous dynamics of the system, which generates these time-series observations can be described by coupled nonlinear ordinary differential equations of the general type used for kinetic modeling:

1 $${\dot{\bf m}}(t) = f({\bf{m}}(t),{\bf{p}}(t)),$$

where **m** and **p** are vectors that denote the metabolite and protein concentrations, as explained above. The function $f:{\Bbb R}^{n + \ell } \to {\Bbb R}^n$ encloses all the information on the system dynamics. Deriving these dynamics from the time series data will be formulated as a supervised learning problem where the function *f* is learned through machine learning methods, which predict the relationship between metabolomics and proteomics concentrations (input features, see Supplementary Fig. S1) and the metabolite time derivative ${\dot{\bf m}}(t)$ (output). In order to provide the training data set for this problem, the metabolite time derivative ${\dot{\tilde{\bf m}}}$ is obtained from the times-series data ${\tilde{\bf m}}(t)$, as shown in Supplementary Fig. S2.

In order to parametrize the machine learning algorithm, the following optimization problem is solved (through scikit-learn, see materials and methods):

#### **Problem 1**

(Supervised Learning of Metabolic Dynamics) *Find a function* *f* *which satisfies*:

2 $$\arg\min_{f} \mathop {\sum}\limits_{i = 1}^q \mathop {\sum}\limits_{t \in T} \left\Vert {f({\tilde{\bf m}}^i[t],{\tilde{\bf p}}^i[t]) - {\dot{\tilde{\bf m}}}^i(t)} \right\Vert^2$$

Solving this problem is equivalent to finding the metabolic dynamics, which best describe the time-series data provided. Once the dynamics are learned we can then predict the behavior of the metabolic pathway by solving an initial value problem (Eqs. (3) and (4).

## Results and discussion

**Fig. 4:**
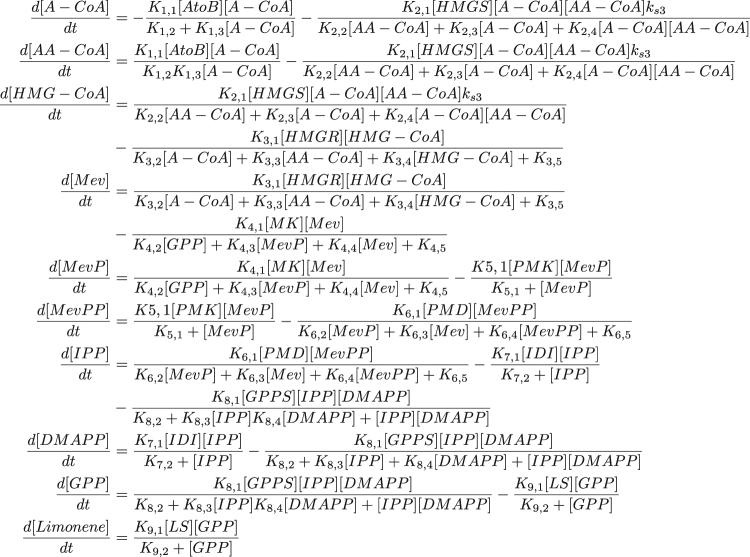
Limonene pathway kinetic Michaelis–Menten model. This kinetic model was compiled from sources in the BRENDA database along with guidance from Weaver et al.^93^ This system is composed of ten nonlinear ordinary differential equations, which describe the concentration for each metabolite in the pathway (see Supplementary Material for details). The dynamics of this model are rich and complex enough to pose a significant challenge to be predicted through machine learning. This model is used in this work to: (1) compare its predictions with machine learning predictions, and (2) generate simulated data sets to check scaling dependencies with the amount of time series used for training of machine learning algorithms. The method presented in this paper focuses on substituting these Michaelis–Menten expressions by machine learning algorithms (see Supplementary Fig. S1). Kinetic constants were left as free parameters when fitting experimental data in Fig. 6

We used the supervised learning method described above (Figs. 1 and 2, Eqs. (1), (2), (3) and (4)) to predict pathway dynamics (i.e., metabolite concentrations as a function of time) from protein concentration data for two pathways of relevance to metabolic engineering and synthetic biology: a limonene producing pathway and an isopentenol producing pathway (Fig. 3). For each pathway we used experimental times-series data obtained from the low and high biofuel producing strains as training data sets, in order to predict the dynamics for the medium producing strains.^66^ Because of the paucity of dense multiomics time-series data sets, we used simulated data sets (Fig. 4) to study the algorithm’s performance as more training data sets (strains) were added.

### Qualitative predictions of limonene and isopentenol pathway dynamics can be obtained with two time-series observations

**Fig. 5:**
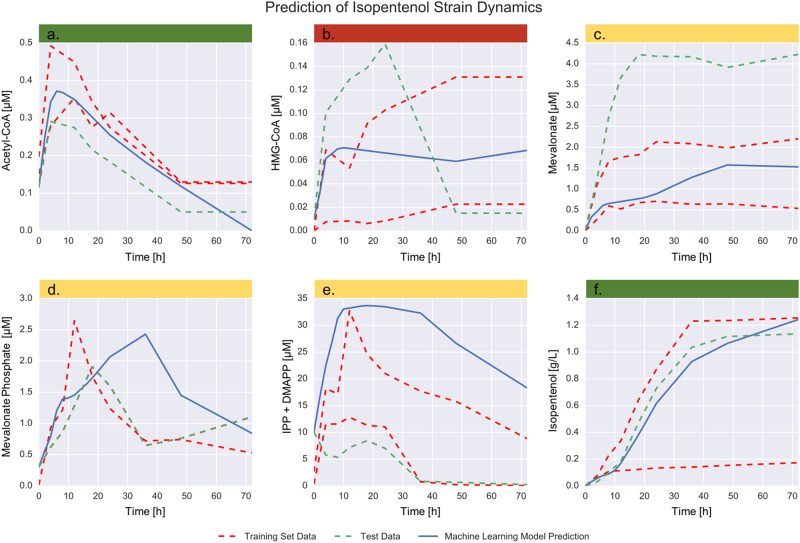
The machine learning method produces acceptable predictions of metabolite time series from proteomics data for the isopentenol producing *Escherichia coli* strain. The measured metabolomics and proteomics data^66^ for the highest and lowest producing strains (training set data, red line) are used to train a model and learn the underlying dynamics (Fig. 2). The model is then tested by predicting the metabolite profiles (blue line) for a strain the model has never seen (medium producing strain, test data in green). A perfect prediction (blue line) would perfectly track the test data set (green line). Interestingly, reasonable qualitative agreement is achieved even with only two time-series (strains) as training data. From a purely quantitative perspective, the average error is high: the total RMSE for the strain predictions is 40.34, which can be translated to 149.2% average error. However, for a couple of metabolites (green color band) the predictions quantitatively reproduce the measured data: Acetyl-CoA and isopentenol (the final product, and most relevant for guiding bioengineering). For some metabolites (mevalonate, mevalonate phosphate and IPP/DMAPP, yellow band), the model qualitatively reproduces the metabolite patterns, missing the scale factor. Only for HMG-CoA does the model fail to predict the metabolite concentration over time both quantitatively and qualitatively (red band)

**Fig. 6:**
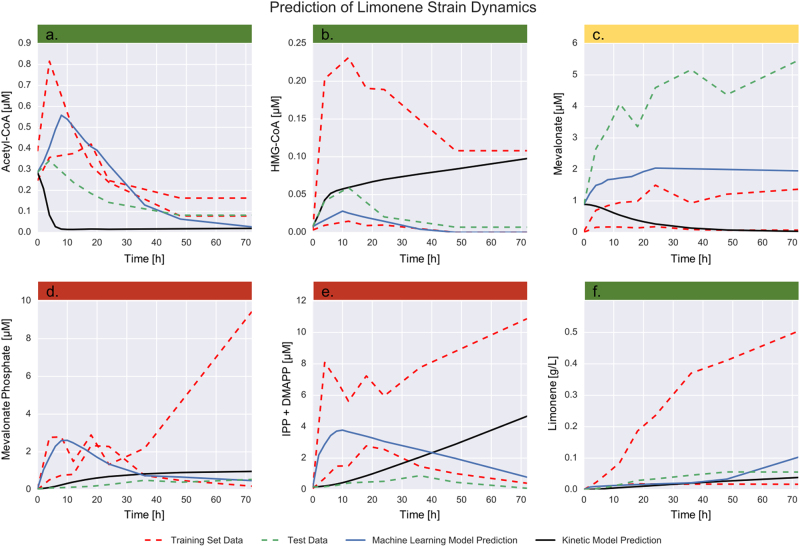
The machine learning method outperforms the handcrafted kinetic model for the limonene producing *E. coli* strain. The only metabolite for which the kinetic model (black line) provides a better fit than the machine learning method (blue line) is mevalonate phosphate, although both methods appear to track limonene (final product) production fairly well. The machine learning approach provides acceptable quantitative fits for Acetyl-CoA, HMG-CoA, and limonene (green band), a qualitative description of metabolite behavior missing the scale factor for mevalonate (yellow band), and fails quantitatively and qualitatively for mevalonate phosphate and IPP/DMAPP (red band). As in Fig. 5, the experimentally measured profiles correspond to high, low and medium producers of limonene. The training sets are the low and high producers (in red) and the model is used to predict the concentrations for the medium producing strain (in green). Kinetic constants for the handcrafted kinetic model in Fig. 4 were left as free parameters when fitting the experimental data

Surprisingly, just two time-series (strains) were enough to train the algorithm to produce acceptable predictions for most metabolites. While the predictions of derivatives from proteomics and metabolomics were quite accurate (aggregate Pearson *R* value of 0.973), any small error in these predictions compounds quickly when solving the initial value problem given by Eqs. (3) and (4). The reason is that predictions for a given time point depend on the accuracy of all previous time points. In spite of these hurdles, the method produced respectable qualitative and quantitative predictions of metabolite concentrations for a strain it has never seen before (Figs. 5 and 6). For some metabolites (33%), the predictions were quantitatively close to the measured profile: Acetyl-CoA (83.4 % error, Fig. 5a) and Isopentenol (43.7 % error, Fig. 5f) for the isopentenol producing pathway; Acetyl-CoA (128.2 % error, Fig. 6a), HMG-CoA (83.9 % error, Fig. 6b) and Limonene (82.9 % error, Fig. 6f) for the limonene producing pathway. For most metabolites (42%), the predictions were off by a scale factor, but they were able to qualitatively reproduce the metabolite behavior. For example, for Mevalonate in the isopentenol producing pathway (Fig. 5c) and mevalonate in the limonene producing pathway (Fig. 6c) the predictions reproduce the initial increase of metabolite concentration followed by a saturation. For IPP/DMAPP (Fig. 5e) or mevalonate phosphate (Fig. 5d) in the isopentenol pathway, the prediction reproduces qualitatively the concentration increase, followed by a peak and a decrease. The prediction of even just this type of qualitative behavior is useful to metabolic engineers in order to obtain an intuitive understanding of the pathway dynamics and design better versions of it. By simulating several scenarios the metabolic engineer can extract qualitative knowledge (e.g., metabolite x seems toxic, or protein y seems regulated by metabolite x) that can lead to testable hypotheses. Finally, in a minority of cases (25%), the predictions are wrong both quantitatively and qualitatively: e.g., HMG-CoA for the isopentenol producing pathway (Fig. 5b), Mevalonate phosphate (Fig. 6d) and IPP/DMAPP (Fig. 6e) for the limonene producing pathway. Interestingly, the predictions for both final products (limonene and isopentenol) fell in the group of quantitatively accurate predictions. This is important because, for the purpose of guiding metabolic engineering, it is the final product predictions that are relevant.

The machine learning approach outperforms a handcrafted kinetic model of the limonene pathway (Fig. 6). A realistic kinetic model of this pathway was built and fit to the data, leaving all kinetic constants as free parameters (Figs. 3 and 4). The kinetic model notably fails to capture the qualitative dynamics for Acetyl-CoA, HMG-CoA, mevalonate, and IPP/DMAPP (Fig. 6a–c, e). More quantitatively, the machine learning model produces an average 130% error (RMSE = 8.42) vs. an average 144% (RMSE = 10.04) for the kinetic model. Hence, even a machine learning model informed by the time series data of just two strains is able to outperform the handcrafted kinetic model, which required domain expertise and significant time investment to construct. The machine learning approach, however, is more easily generalizable and it can be instantly reapplied for a new pathway, host or product by feeding it the corresponding data. Once the predictions were made for the limonene pathway, results for the isopentenol pathway can be obtained easily just by changing the time-series data input. In contrast, in order to make predictions for the isopentenol pathway a new kinetic model would have to be crafted. Kinetic models become more difficult to construct as the size of the reaction network increases and as the knowledge of the relevant network decreases. Additionally, all kinetic relationships must be known or inferred, whereas unknown relationships can be uncovered from data using a machine learning approach. The machine learning approach only requires a sufficient amount of data to disentangle these relationships. Determining how much data is a “sufficient amount” is the goal of the next section.

Interestingly, the model was able to perform well even though the training sets corresponded to pathways which differed in more than just protein levels. This is important because the model is designed to take protein concentrations as input (Fig. 1) in order to predict pathway dynamics, assuming the rest of pathway characteristics to remain the same. This use case covers a wide range of metabolic engineering needs where e.g., promoters and ribosome-binding sites (RBSs) are modified in order to affect the resulting protein concentrations. However, other typically used metabolic engineering strategies include changing a given enzyme in order to access faster or slower catalytic rates (i.e., *k*_cat_). Even though this case was not explicitly contemplated, the model was able to provide good predictions (i.e., I3 was using a HMGR analog form *Staphylococcus aureus* and I2 uses a codon optimized HMGR, see strain description). We hypothesize that *k*_cat_ changes can be renormalized into (and be equivalent to) protein abundance changes. In order to fully address this type of engineering practice, this method may be expanded to include enzyme characteristics as input (besides the proteomics data): *k*_cat_ and *K*_*M*_ constants or even full kinetic characterization curves.

### Increasing the number of strains improves the accuracy of dynamic predictions

We used simulated data to show that predictions improved markedly as more data sets are used for training. Simulated data sets have the advantage of providing unlimited samples to thoroughly test scaling behavior, and allow us to explore a wider variety of types of dynamics than experimentally accessible. Moreover, the dense multiomics time series data sets needed as training data are rare because they are very time consuming and expensive to produce. Since machine learning predictions generally improve as more data is used to train them, we expected our method to improve with the availability of more time series for training. We expected this improvement to be significant since initially only two time-series (strains) were used for training, out of the three available for each product^66^ (the other one was needed for testing). Hence, we used simulated data obtained from using the kinetic model developed for the limonene pathway (Figs. 3 and 4), in order to study: (1) how much predictions improve as more time-series data sets are added and (2) how many time series are needed to guide pathway design effectively (next section). A pool of 10,000 sets of time-series data with different protein profiles was created that shared the same kinetic constants. We fed the machine learning algorithm groups of 2, 10, and 100 times series randomly sampled from this pool in order to study how quickly the algorithm was able to recover the original simulated dynamics. In order to gauge the variability of the predictions (i.e., how predictions change as different training sets are used) as a function of training group size (2, 10, or 100), we repeated the predictions ten times for each training group size.

**Fig. 7:**
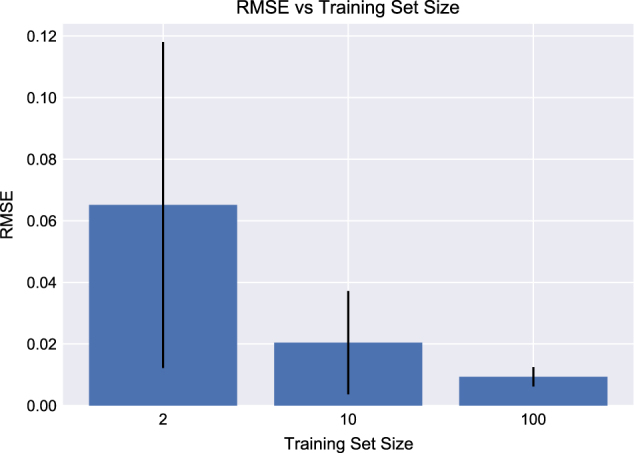
Prediction errors decrease markedly with increasing training set size. As the number of available proteomics and metabolomics times-series data sets (strains) for training increases, the prediction error (RMSE, Eq. (6)) decreases conspicuously. Moreover, the standard deviation of the predictions error (vertical bar) decreases notably as well. The change from 2 to 10 strains is more pronounced that the change from 10 to 100. This fact indicates that it is more productive to do ten rounds of metabolic engineering collecting ten time-series data sets, than a single round collecting 100 time series

**Fig. 8:**
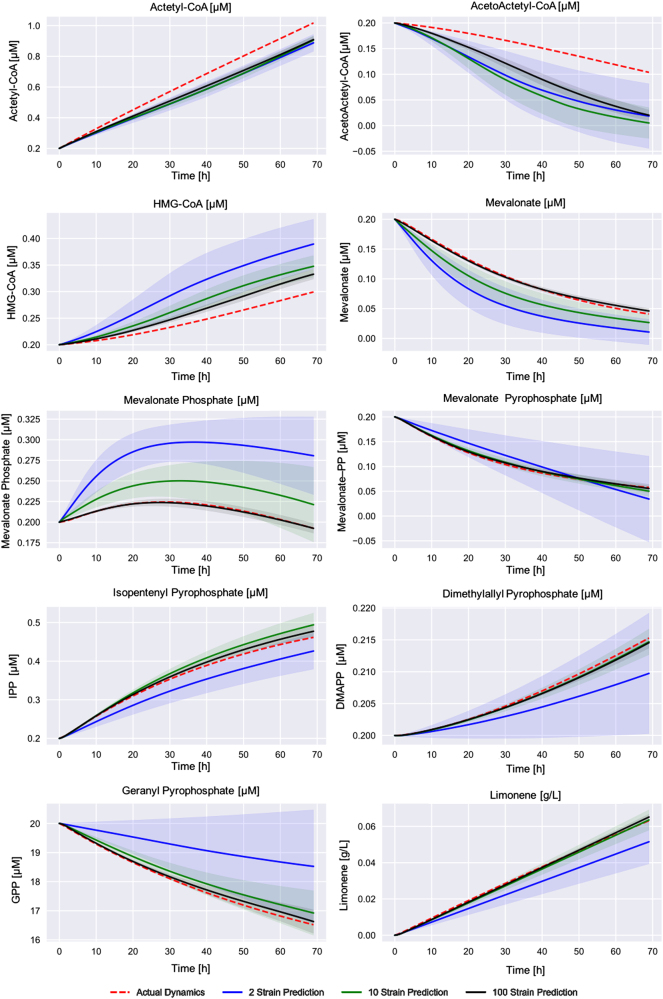
Predictions improve with more training data sets. The machine learning algorithm was used to predict kinetic models for varying sizes of training sets (2, 10, and 100 virtual strains in blue, red and black). Ten unique training sets were used for each size to show prediction variability (transparency) for each training set size. All models converge towards the actual dynamics with the 100 strain models in closest agreement. Standard deviations (shown by the transparency) also decrease markedly as the size of the training set increases

The prediction error (RMSE, Eq. (6)) decreased monotonically as a function of the number of time-series (strains) used to train the algorithm in a nonlinear fashion (Fig. 7). Also, the standard deviation of the predictions significantly decreased with the number of training of data sets (Fig. 8). The standard deviation is an indication of the variability of pathway dynamics predictions due to stochastic effects of the optimization algorithms (e.g., different seeds) and lack of extrapolability from a reduced set of initial protein concentrations. Hence, a predictive model trained with 10 or 100 data sets produces much more robust predictions than a model trained with two data sets. In fact, the high standard deviations observed for models trained on only two data sets explain the prediction variability observed in the previous section due to stochastic effects. Interestingly, there is a limited drop in error and standard deviation from 10 to 100 strains, with the decrease from 2 to 10 being the largest (Fig. 7). This indicates that it is more productive to do ten rounds of engineering collecting ten time-series data set than a single round collecting 100 time series: in this way, ten time series produce accurate enough predictions to pinpoint the desirable part of proteomics of phase space, new strains can be engineered around that space so that new multiomics time series can be obtained around the desirable phase space and optimize for prediction accuracy around that area of phase space. Doing this ten times is more accurate than a single prediction based on 100 time series that may not be close to the ultimately desirable proteomics phase space. Furthermore, it indicates that the results from the previous section would have been much more reliable if only eight time series more had been available for training.

### Model predictions are accurate enough to guide pathway design and produce biological insights

The machine learning predictions do not need to be 100% quantitatively correct to accurately predict the relative ranking of production for different strains. Being able to reliably predict which of several possible pathway designs will produce the highest amount of product is very valuable in guiding bioengineering efforts and accelerating them in order to improve titer, rate, and yield (TRY). These process characteristics are fundamental determinants of economic relevance.^67^

**Fig. 9:**
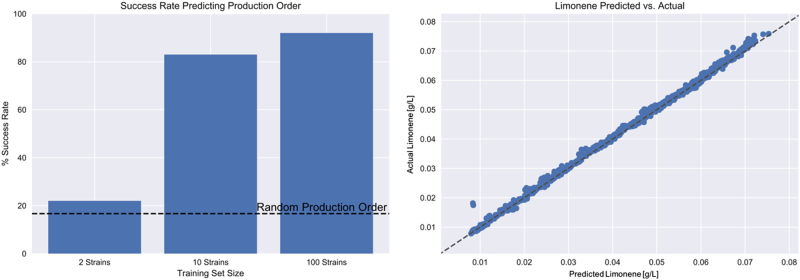
Success rate predicting production ranks increases with training set size. The left panel shows the success rate in predicting the relative production order (i.e., which strain produces most, which one produces least and which one is a medium producer) for groups of three time series (strains) randomly chosen from a pool of 10,000 strains, as a function of training data set size (strains). For 100 data sets, the failure rate to predict the top producer is <10%. For ten data sets the success rate is ~ 80%, which is reliable enough to guide engineering efforts. The horizontal line provides the rate of success (1/6) if order is chosen randomly. The right panel shows that prediction of limonene production is extremely accurate for the case of a training data set comprised of 100 time-series (strains). These data shows that the machine learning model predictions are accurate enough to guide pathway design if enough training data is available

The machine learning algorithm was able to reliably predict the relative production ranking for groups of three randomly chosen strains (highest, lowest, and medium producer, mimicking the available experimental data) chosen from the pool of 10,000 time-series data sets mentioned above (Fig. 9, left panel). The success rate depended critically on the number of data sets available for training: starting at 22% for only two strains up to 92% for 100 training sets. For ten strains the success rate is ~ 80%, which is reliable enough to practically guide metabolic engineer efforts to improve TRY. For models trained using 100 time series, the prediction errors were minimal (Fig. 9, right panel).

**Fig. 10:**
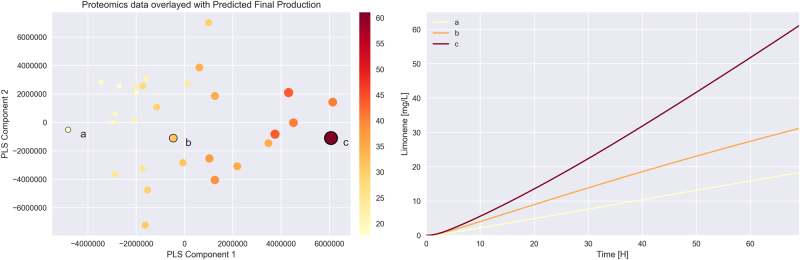
The ML approach can be used to produce biological insights. The left panel shows the final position in the proteomics phase space (similarly to the PCAP^68^ approach) for 50 strains generated by the ML algorithm by learning from the Michaelis–Menten kinetic model (Fig. 4) used as ground truth. Final limonene production is given by circle size and color. The PLS algorithm finds directions in the proteomics phase space that best align with increasing limonene production (component 1). Traveling in proteomics phase space along that direction (which involves overexpression of LS and underexpression of AtoB, PMD, and Idi, see Table S2) creates strains with higher limonene production. The ML approach not only produces biological insights to increase production but also predicts the expected concentration as a function of time for limonene and all other metabolites, generating hypotheses that can be experimentally tested (right panel)

Biological insights can be generated by using the machine learning (ML) model to produce data in substitution of bench experiments. For example, similarly to principal component analysis of proteomics (PCAP^68^), we can use the ML simulations to determine which proteins to over/underexpress, and for which base strain, in order to improve production (Fig. 10). Proteins LS, AtoB, PMD, and Idi are the most important drivers of production in the case of limonene: changing protein expression along the principal component associated with them increases limonene creation (Fig. 10, left panel). Furthermore, this approach provides expected behavior for all metabolites in the pathway, providing hypotheses that can be tested experimentally (Fig. 10, right panel).

### Data constraints are significant but surmountable

Since the ML approach is purely data-based, data quantity and quality concerns are of paramount importance. Data quantity concerns involve both the availability of enough time series as well as time points sampled in each time series.

The training set used here^66^ is one of the largest data sets characterizing a metabolically engineered pathway at regular time intervals through proteomics and metabolomics. There are no larger data sets that include: time series, several types of omics data, more than seven time points, and several strains. For example: the *E. coli* multiomics database^69^ has proteomics and metabolomics data for several strains, but no time series; Ma et al.^70^ report proteomics and metabolomics data but only one time series with fewer time points (5 instead of 7); Yang et al.^71^ only provide one time series and only one time point for proteomics; Doerfler et al.^72^ and Dyar et al.^73^ only provide time-series metabolomics data; Patel et al.^74^ does not combine metabolomics and proteomics and data download was disabled at the time of testing; the DOE kbase^75^ focuses on genomics and does not have any time-series proteomics or metabolomics publicly available; and the Experiment Data Depot^76^ does not have any studies surpassing this one in terms of data points and strains.

In order to get enough pairs of derivatives and proteomics and metabolomics data to train ML algorithms (Supplementary Fig. S1), we have used data augmentation (filtering and interpolation, Fig. 2 and Supplementary Fig. S2), expanding the initial seven time points to 200 by just assuming continuity in the multiomics data (a reasonable assumption in our experience). It would be desirable to have more time points available, so as to not to depend on these data augmentation techniques. However, data sets including more time points are nonexistent for physical, biological, and economical reasons. Every time a sample is taken for -omics analysis, the volume in the culture flask diminishes and, if the total sampled volume is comparable to the total volume, it may significantly affect the strain physiology. Since taking excessive samples may affect measurements, and these coupled omics analysis are expensive and require specialized personal, it is not surprising that the maximum amount of time points we have seen is ~ 7. Another reason more time points have not been typically collected is that experts in multiomics data collection consider this sampling rate to fully capture the physiology of strains based on previous experience.^77,78^ The fact that we are able to produce reasonable predictions for a third time series that the algorithm has never seen before (test strain) validates this, and the multiomics data continuity assumption.

### Future work

The application of machine learning to synthetic biology will hopefully open up new avenues of research as well as accelerate the adoption of modeling in bioengineering and beyond. This work is a first step demonstrating that a purely data-driven approach can fruitfully predict biological dynamics. There are plenty of possible ways to improve it.

An obvious first step involves adding other supervised learning techniques to improve predictions. The current approach uses tree-based pipeline optimization tool (TPOT) to combine, through genetic algorithms, 11 different machine learning regressors and 18 different preprocessing (feature selection) algorithms. New supervised learning techniques can be added to this approach by adding them to the scikit-learn library.^79^ TPOT will automatically test them and use them if they provide more accurate predictions than the techniques used here. Among the most popular algorithms for ML are deep-learning (DL) techniques based on neural networks. However, the small size of the available data sets for this study limited the use of machine learning techniques to classical methods. Modern DL techniques typically require orders of magnitude more data than was used in this study (~ 1000 strains as a starting point). While this amount of data is currently cost prohibitive, it is a worthy goal to move towards DL: these methods have demonstrated super human performance across a variety disciplines. These include, for example, image labeling tasks, in which humans have evolved proficiency. In domains where humans are less capable, such as the dynamical system characterization considered here, super human performance should be substantially easier to achieve. The payoff would involve radically improving engineering outcomes by making the predictability of complex biological systems proportional to the quantity of input data.

An often posed question is whether mechanistic insights can be inferred from ML approaches. While this is not trivial, there are a couple of possibilities for this inference: (1) for any particular ML model that produces good fits, the most relevant features (i.e. protein x has the highest weight in determining y molecule concentration) provides a prioritized list of putative mechanistically linked parts that can be further investigated. (2) the ML model can be used as a surrogate for high-throughput experiments to derive mechanistic biological insights (Fig. 10). Another example of this last approach would involve studying toxicity by adding cell biomass (through optical density, OD) to the measurements and simulate for a variety of scenarios (protein inputs) the correlation between OD and all metabolites: a negative correlation would signal putative toxic metabolites.

It is instructive, however, to pause and reflect on the drive to find mechanisms. Mechanisms offer a causally related set of processes and parts that produce the observed phenomena. Understanding these processes, parts, and causal relations produces a knowledge that can indeed be transferred to predict the behavior of different systems (pathways, strains, products. etc) where the same mechanism is involved. However, biology has been particularly inefficient in making predictions of complex systems from known and tested mechanisms. If our final goal is to predict new biological systems, it may be more successful to look into ML techniques such as transfer learning.^80^ These techniques tackle directly the challenge of predicting systems based on data originated in related systems without the need to delve into mechanisms. Having said this, there is not doubt that the most desirable outcome is a model that is both predictive and mechanistic, but if we are to do without one of these characteristics, the mechanistic knowledge may be the least immediately useful for current bioengineering.

Infusing prior knowledge into the ML approach is a related possible future research avenue. Currently, our method does not constrain the vector fields that are learned using any biological intuition. There are often biological facts known about these dynamical systems that could be use to improve the performance of our method. Specifically, genome-scale stoichiometric constraints could provide guarantees that the resulting system dynamics conserve mass and conform to our prior knowledge about the organism.

Since the procedure outlined here requires little prior biological knowledge, it is enticing to imagine extending this method for use with different data inputs or other types of applications. An obvious extension is to use transcriptomics data as input. Given the current exponential increase in sequencing capabilities, transcriptomics data is more amenable to high-throughput production than proteomics and metabolomics data. Our biological intuition says that transcriptomics data should be less informative than proteomics, but it is surely interesting to explore whether that can be countered with more time series (and how many). It would also be of interest to use the ML method to predict proteomics in addition to metabolomics time series. Another logical proposition is to expand this method to encompass genome-scale multiomics data. We surmise that the extra predictive capabilities of the machine learning with respect to the Michaelis–Menten approach proceed, in part, from indirectly accounting for host metabolism effects through proxies (e.g., metabolites or proteins that are affected indirectly by host metabolism). Hence, we expect more comprehensive metabolomics and proteomics (as well as transcriptomics) data sets to increase the method predictive accuracy. A more intriguing and bold endeavor would be to apply this method to predict microbial community dynamics using metaproteomics and metabolite concentration data as inputs. There is nothing in this approach that constrains it to intracellular pathway prediction and microbiome research, and industry has a definite need for increased predictive power.^81^ Finally, the incoming availability of dense multiomics data sets for human metabolism provides an alluring target.^82,83^

## Conclusion

We have demonstrated that it is possible to use a pure machine learning approach to qualitatively predict pathway dynamics. This approach, using only two time-series (strains) as training data, was able to outperform in predictive power a classical Michaelis–Menten kinetic model. Unlike traditional kinetic modeling, we do not need to assume any particular interaction (e.g., allosteric regulation), but we give full freedom to the system to implicitly choose the ones that best predict the experimental data. Furthermore, we were able to produce predictions that, although not fully quantitatively accurate, are precise enough to drive design decisions given enough data: production rankings can be predicted. The ability to predict the pathway dynamics is of significant interest to metabolic engineers and synthetic biologists, since it allows for building an intuitive understanding of the pathway that can produce testable hypotheses (yield increase, compound toxicity). This method is also an example of the benefit of targeting the prediction of derivatives using machine learning in order to predict dynamic processes.

We have also shown that the machine learning approach improves markedly by using more time series (strains) as training sets, and used simulated data to estimate the number of time series required to guide engineering. Although the training set used here^66^ is one of the largest data sets characterizing a metabolically engineered pathway at regular time intervals through proteomics and metabolomics, it is barely sufficient to train machine learning algorithms. Another limitation of this work involves only being able to test the method with two pathways, which are the only ones for which dense time-series multiomic data sets are available. These limitations justify future efforts directed at methodic collection of large time-series data sets as enabled by multiomic pipelines,^84–87^ since this method provides a systematic method to productively leverage those data. Moreover, coupled with recent developments providing real-time metabolomics capabilities,^88^ this method opens the alluring possibility of real-time prediction and control of biological pathways.

These results open the door to a data-centric approach to predicting metabolism that can greatly benefit the biotech and synbio industries, much necessitated of predictive power in order to enable reliable production.^13,89^ This approach is agnostic as to the pathway, host or product used, and can be systematically applied, as we have shown. Unlike previous approaches,^66^ it can systematically leverage proteomics and metabolomics data in a fashion that increases accuracy as more data is available. Besides being of immediate practical utility for bioengineering, this approach can be used as a first step in improving mechanistic kinetic models by pinpointing the most relevant machine learning features for accurate predictions, that can then be followed up by further experiments in order to obtain a mechanistic understanding of the reasons for their predictive power.

This work shows that, given sufficient data, the dynamics of complex coupled nonlinear systems relevant to metabolic engineering can be systematically learned.

## Materials and methods

### Learning system dynamics from time-series data

The core of this method consists in using machine learning methods to predict the functional relationship between the metabolite derivative and proteomics and metabolomics data, substituting the Michaelis–Menten relationship (Eq. (1), Supplementary Fig. S1 and Fig. 4). The first step involves creating a training set comprising sets of proteomics and metabolomics data and their corresponding derivatives (Supplementary Fig. S1). This entails computing the derivatives of the metabolite concentration time-series data. Because the time-series data is subject to measurement noise, the derivatives must be carefully estimated. The second step involves finding the best performing regression technique, among the many possibilities available.^79^ Finally, once the best prediction algorithm is found and cross-validated, we can use it to predict metabolite concentrations given initial time points. The complete code to implement these steps is provided in github (see below).

#### Construction of the training data set

In order to train a machine learning model, a suitable training set must be created. We expect the trained machine learning model to take in metabolite and protein concentrations at a particular point in time and return the derivative of the metabolite concentrations at the same time point (Supplementary Fig. S1). The observations provide us with the inputs to the model, ${\tilde{\bf m}}^i[t]$ and ${\tilde{\bf p}}^i[t]$. In order to have examples of correct outputs for supervised learning we have to estimate the derivatives of the metabolite time series data, ${\dot{\tilde{\bf m}}}^i(t)$ (Supplementary Fig. S2).

Naively computing the derivative of a noisy signal will amplify the noise and make the result unusable. Derivatives of noisy signals, like those obtained from experiments, require extra effort to estimate. In order to estimate the time derivatives on time series of real data obtained from Brunk et al.^66^ accurately, we apply a Savitzky–Golay^90^ filter to the noisy time-series data to find a smooth estimate of the data (Supplementary Fig. S2). This smooth function estimate can then be used to compute a more accurate estimate of the derivative. We compute the derivative estimate of the signal using a central difference scheme from the filtered experimental data. Specifically, the Savitzky-Golay filter is used with a filter window of 7 and a polynomial order of 2. The derivative estimate, ${\dot{\tilde{\bf m}}}^i(t)$, is computed for all time points in *T* and time series *i*. This results in a training example associated with each time point in every time series.

This work assumes that all relevant metabolites are measured and that the system has no unmeasured memory states. In other words, the present set of metabolite and protein measurements completely determines the metabolite derivatives at the next time instant. If this assumption does not hold practically, a limited time history of proteins and metabolites can be used to predict the derivative at the next time instant. We observe that, for the specific pathways used in this paper, this assumption produces good predictions.

#### Model selection

The model selection process used a meta-learning package in python called TPOT.^91^ Once the training data set is established, a machine learning model must be selected to learn the relationship between input and outputs (Supplementary Fig. S1). TPOT uses genetic algorithms to find a model with the best cross-validated performance on the training set. Cross validation techniques are used to score an initial set of models. The best performing models are mated to form a new population of models to test. This process is repeated for a fixed number of generations and the best performing model is returned to the user.^92^ If desired, the search space for model selection can be specified before execution of the TPOT regressor search. This might be done to prune models that require long training times or to select only models that have desirable properties for the problem under consideration. Specifically, we used TPOT to select the best pipelines it can find from the scikit-learn library^79^ combining 11 different regressors and 18 different preprocessing algorithnms. This model selection process is done independently for each metabolite (Supplementary Table S1). After TPOT determines the optimal models associated with each metabolite, they are trained on the data set of interest and are ready for use to solve Eqs. (3) and (4). Models with the lowest tenfold cross-validated prediction root mean squared error were selected. In this way, the best validated models were selected for use.

After automated model selection via TPOT, we manually evaluated each model based on its accuracy in predicting metabolite derivatives given protein and metabolite concentration at a given time point (Supplementary Fig. S1). Each data set used for model fitting was split into training and test sets ten times using the shuffle split methodology implemented in scikit-learn.^79^ After the model was fit, predictions on both the training and test sets were computed for each metabolite model and their predictive ability quantified through a Pearson R^2^ coefficient (e.g., Supplementary Fig. S3).

#### Using the model

Once the models are trained, we can use them to predict metabolite concentrations by solving the following initial value problem using the same function *f* that was learned in Eqs. (1) and (2):

3 $${\dot{\mathbf m}} = f({\mathbf{m}},{\tilde{\mathbf p}})$$

4 $${\mathbf{m}}(t_0) = {\tilde{\mathbf m}}(t_0)$$

This problem is solved by integrating the system forward in time numerically. As a general purpose numerical integrator, we used a Runga Kutta 45 implementation.

### Data set curation and synthesis

Two different data sets were used in this work. The first is an experimental data set curated from a previous publication,^66^ comprising three proteomic and metabolomic time-series (strains) from an isopentenol producing *E. coli* and three time-series (strains) from limonene producing *E. coli*. The second data set involves computationally simulated data from a kinetic model of the limonene pathway, which is used to test how the method performance scales with the number of time series used.

#### Description of a real time-series multiomics data set

Proteomics and metabolomics data for two different heterologous pathways engineered into *E. coli* were available from Brunk et al.^66^ There are three (high, medium, and low production) variants for strains which produce isopentenol and limonene, respectively. All strains were derived from *E. coli* DH1. The low and high-producing strain for each pathway were used to predict the medium production strain dynamics by solving Eqs. (3) and (4).

The isopentenol producing strains (I1, I2 and I3) were engineered to contain all of the proteins required to produce isopentenol from acetyl-CoA as (Fig. 3). I1 is the unoptimized strain containing the naive variants of each protein in the pathway. I2 differs from the base strain I1 in that it contains a codon optomized HMGR enzyme along with the positions of PMK and MK swapped on its operon. I3 uses an HMGR homolog from *Staphylococcus aureus*. Limonene producing strains (L1, L2, and L3) produce limonene from acetyl-CoA (Fig. 3). L1 is the unoptimized strain with the naively chosen variants for each protein in the pathway. It is a two plasmid system where the lower and upper parts of the pathway are split between both constructs. L2 is a DH1 variant that contains the entire limonene pathway on a single plasmid. L3 is another two plasmid strain where the entire pathway is present on the first plasmid, and the terpene synthases are on a second plasmid for increased expression. Starting at induction, each strain had measurements taken at seven time points during fermentation over 72 h. At each time point pathway, metabolite measurements and pathway protein measurements were collected. Further details on these strains and experimental design can be seen in the original publication.^66^

#### Data augmentation through filtering and interpolation

In the training set each time series contains seven data points. These are too sparse to formulate accurate models. To overcome this a data augmentation scheme is employed where seven time points from the original data are expanded into 200 for each strain. This is done by smoothing the data with a Savitzky-Golay filter and interpolating over the filtered curve (Fig. 2 and Supplementary Fig. S2). When predicting the dynamics of a medium production strain from high and low producing strains, we performed model selection by scoring each model using tenfold cross validation and a Pearson R^2^ metric on two data augmented training strains.

#### Development of realistic kinetic models

In order to study the scaling of performance as more training sets were added, a realistic and dynamically complex model of the mevalonate pathway was developed from known interactions extracted from the literature (Figs. 3 and 4). The dynamic model is implemented with Michaelis–Menten like kinetics and is a 10 state coupled nonlinear system. Complete details of this kinetic model are available in the Supplementary Material. The objective was to create a realistic model, relevant to metabolic engineering, for which learning the system dynamics is a non-trivial task on par with the difficulty of learning real system dynamics from experimental data.

#### Generation of a simulated data set

The kinetic model described above was used to create a set of virtual data time-series (strains). The kinetic model coefficients were chosen to be close to values reported in the literature while maintaining a non-trivial dynamic behavior.

A virtual strain is created by first generating a pathway proteomic time series. This is done by randomly choosing three coefficients for each protein (*k*_*f*_, *k*_*m*_, *k*_*l*_), which specify a leaky hill function. The hill function was chosen because it models the dynamics of protein expression from RNA accurately. This leaky hill function specifies the protein measurements for each time point and is defined in the equation below:

5 $$\tilde p(t) = \frac{{k_{f}\;t}}{{k_m + t}} + k_l$$

Once all protein time series are specified, they are used in conjunction with the kinetic coefficients to solve the initial value problem in Eqs. (3) and (4) in order to determine the time series of metabolite concentrations. The resulting data set is a collection of time-series measurements of different strain proteomics and metabolomics. All strains use the same kinetic parameters and differential equations to generate the metabolomics measurements. The code used to generate this data can be found in the github repository, as well.

### Fitting the Michaelis–Menten kinetic model

To compare the handcrafted kinetic model with the data-centric machine learning methodology, the parameters of the kinetic model were fit to strain data. To find the best fit we used a differential evolution algorithm implemented in scipy. This global optimizer was chosen because its convergence is independent of the initial population choice and it tends to need less parameter tuning than other methods. All kinetic parameters were constrained to be between 10^−12^ and 10^9^. This large range of acceptable parameter values allowed for maximum flexibility of the kinetic model to describe the data.

### Evaluation of model performance for time series

Dynamical prediction was tested on a held back strain that the model did not use in training. When using the experimental data sets,^66^ the medium titer strains were held back for testing. When using simulated data, a random strain from the data set was selected. For each time series, agreement between predictions and test data was assessed by calculating the root mean squared error (RMSE) of the predicted trajectories:

6 $${\mathrm R}{\mathrm M}{\mathrm S}{\mathrm E} = \sqrt {\frac{1}{n}\mathop {\sum}\limits_{j = 1}^n \mathop {\int}\limits_{t_0}^{t_f} \left( {\bar m_j(t) - m_j(t)} \right)^2dt}$$

where $\bar m_j(t)$ is the interpolation of the actual metabolite concentration of metabolite *j* at time *t* (Supplementary Fig. S2), and $m_{j}(t)$ is the prediction obtained from solving Eqs. (3) and (4).

### Biological insight analysis

In order to showcase how biological insights can be derived (Fig. 10), we trained the ML model using 50 proteomics and metabolomics time series, using the Michaelis–Menten kinetic model as ground truth. Another 50 proteomics time series were held back as a test data set. Each metabolite time series was predicted using the machine learning model and the associated proteomic time series. The final time point proteomics and final production were collected for each predicted strain. The final time point proteomics data was plotted in two dimensions with a basis selected by performing a partial least squares [PLS] regression between the proteomics and final production data. These first basis vector from a PLS regression is the direction that explains the most covariance between the proteomics data and production data. The PLS regression was implemented by and used from scikit-learn.

### Code availability

Code is available at the following code repository: https://github.com/JBEI/KineticLearning.

### Data availability

All data was obtained from Brunk et al.,^66^ and is also available at the code repository: https://github.com/JBEI/KineticLearning.

## Electronic supplementary material

Supplementary Material

## Electronic supplementary material

**Supplementary information** accompanies the paper on the *npj Systems Biology and Applications* website (10.1038/s41540-018-0054-3).
