# Supplementary material for: A machine learning approach to predict metabolic pathway dynamics from time-series multiomics data
Source: NPJ Syst Biol Appl. 2018 May 29;4:19. doi: 10.1038/s41540-018-0054-3 (PMC5974308; doi:10.1038/s41540-018-0054-3)
Supplement: Supplementary file 1 — Supplementary Material [file 41540_2018_54_MOESM1_ESM.pdf]

# Supplementary Material

## 1 Development of a Kinetic Model for Limonene Synthesis

Below is a detailed account of each reaction in the limonene pathway including likely inhibiting metabolites. When put together this provides a solid starting point for a mechanistic metabolic model for limonene production.

### 1.1 Reaction 1

Acetyl-CoA is converted to acetoacetyl-CoA using acetyl-CoA acetyltransferase (AtoB) using a ping-pong mechanism. This enzyme is inhibited by

$$2\,\text{acetyl-CoA} \xrightarrow{\text{AtoB}} \text{CoA} + \text{acetoacetyl-CoA}$$

The ping pong mechanism of this reaction is illustrated as

$$\mathrm{s} + \mathrm{e} \underset{k_{r1}}{\overset{k_{f1}}{\rightleftharpoons}} \mathrm{c} \xrightarrow[k_{c1}]{\nearrow \mathrm{p}_1} \mathrm{e}^* + \mathrm{s} \underset{k_{r2}}{\overset{k_{f2}}{\rightleftharpoons}} \mathrm{c}^* \xrightarrow[k_{c2}]{\nearrow \mathrm{p}_2} \mathrm{e}$$

The mass action law describing this mechanism of reaction 1 ($R_1$) can be described by the following system of ordinary differential equations

$$R_1 \begin{cases} \dot{s} = k_{r1}c + k_{r2}c^* - k_{f1}se - k_{f2}se^* \\ \dot{e} = k_{r1}c + k_{c2}c^* - k_{f1}se \\ \dot{c} = k_{f1}se - k_{r1}c - k_{c1}c \\ \dot{p}_1 = k_{c1}c \\ \dot{e}^* = k_{c1}c + k_{r2}c^* - k_{f2}se^* \\ \dot{c}^* = k_{f2}se^* - k_{r2}c^* - k_{c2}c^* \\ \dot{p}_2 = k_{c2}c^* \end{cases}$$

Using the quasi-steady state assumption this can be rewritten in a Michealis Menten formulation. The resulting equation which describes the pathway product in terms of substrate concentrations is given by

$$\dot{s} = -\frac{K_1 e_0 s}{K_2 + K_3 s}$$

$$\dot{p}_2 = \frac{K_1 e_0 s}{K_2 + K_3 s}$$

where

$$K_1 = k_{c1}k_{c2}k_{f1}k_{f2}$$
$$K_2 = k_{c1}k_{c2}k_{f2} + k_{c1}k_{f1}(k_{c2} + k_{r2}) + k_{c2}k_{f2}k_{r1}$$
$$K_3 = (k_{c1} + k_{c2})k_{f1}k_{f2}$$

## 1.2 Reaction 2

Acetoacetyl-CoA is converted to HMG-CoA by HMGS using a 3 step ping pong mechanism reaction involving an acylation, a condensation, and a hydrolysis [10]. The reaction is given by:

$$\text{acetyl-CoA} + \text{acetoacetyl-CoA} + \text{H}_2\text{O} \xrightarrow{\text{HMGS}} \text{HMG-CoA} + \text{CoA}$$

The three step ping pong mechanism is as shown below

$$\mathrm{s_1} + \mathrm{e} \underset{k_{r1}}{\overset{k_{f1}}{\rightleftharpoons}} \mathrm{c} \xrightarrow[k_{c1}]{\nearrow \mathrm{p_1}} \mathrm{e^*} + \mathrm{s_2} \underset{k_{r2}}{\overset{k_{f2}}{\rightleftharpoons}} \mathrm{c^*} + \mathrm{s_3} \xrightarrow[k_{c2}]{\nearrow \mathrm{p_2}} \mathrm{e}$$

where $p_1$ is CoA and $p_2$ is HMG-CoA. The resulting differential equations for this system are given by:

$$R_2 \begin{cases} \dot{s}_1 = k_{r1}c - k_{f1}s_1 e \\ \dot{e} = k_{r1}c + k_{c2}s_3 c^* - k_{f1}s_1 e \\ \dot{c} = k_{f1}s_1 e - k_{r1}c - k_{c1}c \\ \dot{p}_1 = k_{c1}c \\ \dot{e}^* = k_{c1}c + k_{r2}c^* - k_{f2}s_2 e^* \\ \dot{s}_2 = k_{r2}c^* - k_{f2}s_2 e^* \\ \dot{c}^* = k_{f2}s_2 e^* - k_{c2}s_3 c^* - k_{r2}c^* \\ \dot{s}_3 = -k_{c2}s_3 c^* \\ \dot{p}_2 = k_{c2}c^* \end{cases}$$

Assuming quasi-steady state and constant $H_2O$ concentration yields the Michaelis Menten equations

$$\dot{s}_1 = -\frac{K_1 e_0 s_1 s_2 s_3}{K_2 s_2 + K_3 s_1 + K_4 s_1 s_2}$$

$$\dot{s}_2 = -\frac{K_1 e_0 s_1 s_2 s_3}{K_2 s_2 + K_3 s_1 + K_4 s_1 s_2}$$

$$\dot{p}_2 = \frac{K_1 e_0 s_1 s_2}{K_2 s_1 + K_3 s_2 + K_4 s_1 s_2}$$

where

$$\begin{aligned} K_1 &= k_{c1} k_{c2} k_{f1} k_{f2} \\ K_2 &= k_{c1} k_{c2} k_{f2} s_3 + k_{c2} k_{f2} k_{r1} s_3 \\ K_3 &= k_{c1} k_{c2} k_{f1} s_3 + k_{c1} k_{f1} k_{r2} \\ K_4 &= k_{c1} k_{f1} k_{f2} + k_{c2} k_{f1} k_{f2} s_3 \end{aligned}$$

### 1.3 Reaction 3

Guessing an ordered sequential reaction mechanism with two competitive inhibitors with respect to HMG-CoA. This reaction is inhibited by acetyl-CoA and acetoacetyl-CoA [7]. Because of similarity in substrate and inhibitor structure I am assuming that it is competitive with respect to HMG-CoA.

$$\text{HMG-CoA} + \text{NADPH} \xrightarrow{\text{HMGR}} \text{Mevalonate} + \text{NADP+}$$

$$\mathrm{s_1} + \mathrm{e} \underset{k_{r1}}{\overset{k_{f1}}{\rightleftharpoons}} \mathrm{c_1} + \mathrm{s_2} \underset{k_{r2}}{\overset{k_{f2}}{\rightleftharpoons}} \mathrm{c_2} \xrightarrow{k_{c1}} \mathrm{c_3} \underset{k_{r3}}{\overset{k_{f3}}{\rightleftharpoons}} \mathrm{c_4} + \mathrm{p_1} \underset{k_{r4}}{\overset{k_{f4}}{\rightleftharpoons}} \mathrm{e} + \mathrm{p_2}$$

$$R_3 \begin{cases} \dot{s}_1 = k_{r1} c_1 - k_{f1} s_1 e \\ \dot{e} = k_{r1} c_1 - k_{f1} s_1 e + k_{f4} c_4 - k_{r4} p_2 e - k_{fi1} e i_1 - k_{fi2} e i_2 \\ \dot{c}_1 = k_{f1} s_1 e - k_{r1} c_1 - k_{f2} s_2 c_1 + k_{r2} c_2 \\ \dot{s}_2 = k_{r2} c_2 - k_{f2} s_2 c_1 \\ \dot{c}_2 = k_{f2} s_2 c_1 - k_{r2} c_2 - k_{c1} c_2 \\ \dot{c}_3 = k_{c1} c_2 + k_{r3} p_1 c_4 - k_{f3} c_3 \\ \dot{c}_4 = k_{f3} c_3 - k_{r3} p_1 c_4 + k_{r4} p_2 e - k_{f4} c_4 \\ \dot{c}_5 = k_{fi1} e i_1 - k_{ri1} c_5 \\ \dot{c}_6 = k_{fi2} e i_2 - k_{ri2} c_6 \\ \dot{p}_1 = k_{f3} c_3 - k_{r3} p_1 c_4 \\ \dot{p}_2 = k_{f4} c_4 - k_{r4} p_2 e \\ \dot{i}_1 = -k_{fi1} e i_1 + k_{ri1} c_5 \\ \dot{i}_2 = -k_{fi2} e i_2 + k_{ri2} c_6 \end{cases}$$

Assuming a roughly constant ratio of NADPH to NADP+ and quasi-steady state enzyme balance we can write these equations more simply as:

$$\dot{s}_1 = -\frac{K_1 e_0 s}{K_2 i_1 + K_3 i_2 + K_4 s + K_5} \tag{1}$$

$$\dot{p}_1 = \frac{K_1 e_0 s}{K_2 i_1 + K_3 i_2 + K_4 s + K_5} \tag{2}$$

### 1.4 Reaction 4

Mevalonate Kinase Proceeds via an ordered sequential mechanism. Where mevalonate binds to the enzyme first, followed by ATP. After catalysis, phosphomevalonate is released followed by ADP [2, 9]. (Need to check if these are ordered in all species – looks like hog and s.cerevisiea enzymes are quite different)

$$\text{ATP} + \text{mevalonate} \xrightarrow{\text{MK}} \text{ADP} + \text{phosphomevalonate}$$

The ordered sequential mechanism for Mevalonate Kinase

$$\mathrm{s}_1 + \mathrm{e} \underset{k_{r1}}{\overset{k_{f1}}{\rightleftharpoons}} \mathrm{c}_1 + \mathrm{s}_2 \underset{k_{r2}}{\overset{k_{f2}}{\rightleftharpoons}} \mathrm{c}_2 \xrightarrow{k_{c1}} \mathrm{c}_3 \underset{k_{r3}}{\overset{k_{f3}}{\rightleftharpoons}} \mathrm{c}_4 + \mathrm{p}_1 \underset{k_{r4}}{\overset{k_{f4}}{\rightleftharpoons}} \mathrm{e} + \mathrm{p}_2$$

$$R_4 \begin{cases} \dot{s}_1 = k_{r1}c_1 - k_{f1}s_1e \\ \dot{e} = k_{r1}c_1 - k_{f1}s_1e + k_{f4}c_4 - k_{r4}p_2e \\ \dot{c}_1 = k_{f1}s_1e - k_{r1}c_1 - k_{f2}s_2c_1 + k_{r2}c_2 \\ \dot{s}_2 = k_{r2}c_2 - k_{f2}s_2c_1 \\ \dot{c}_2 = k_{f2}s_2c_1 - k_{r2}c_2 - k_{c1}c_2 \\ \dot{c}_3 = k_{c1}c_2 + k_{r3}p_1c_4 - k_{f3}c_3 \\ \dot{c}_4 = k_{f3}c_3 - k_{r3}p_1c_4 + k_{r4}p_2e - k_{f4}c_4 \\ \dot{c}_5 = k_{fi1}ei_1 - k_{ri1}c_5 \\ \dot{c}_6 = k_{fi2}ei_2 - k_{ri2}c_6 \\ \dot{p}_1 = k_{f3}c_3 - k_{r3}p_1c_4 \\ \dot{p}_2 = k_{f4}c_4 - k_{r4}p_2e \\ \dot{i}_1 = -k_{fi1}ei_1 + k_{ri1}c_5 \\ \dot{i}_2 = -k_{fi2}ei_2 + k_{ri2}c_6 \end{cases}$$

GPP and FPP are both competitive inhibitors of MK with respect to ATP [4]. In the Streptococcus pneumoniae homolog of mevalonate kinase, diphosphomevalonate (DPM) is an noncompetitive inhibitor with respect to both substrates. DPM binds at an allosteric site, and inhibition cannot be overcome by an increasing substrate concentration. [1].

The resulting Michealis Menten Equations Assuming ATP and ADP are roughly constant and two inhibitors

$$\dot{s}_1 = -\frac{K_1 e_0 s}{K_2 i_1 + K_3 i_2 + K_4 s + K_5} \tag{3}$$

$$\dot{p}_1 = \frac{K_1 e_0 s}{K_2 i_1 + K_3 i_2 + K_4 s + K_5} \tag{4}$$

### 1.5 Reaction 5

Phosphomevalonate Kinase proceeds with a random sequential bi-bi mechanism in the S. pneumoniae homolog. [8]. The enzyme is kinetically characterized in [3] for S. Cerevisiae, however, in the paper they claim that it would be superior to use the better characterized enzyme in S. pneumoniae.

$$\text{ATP} + \text{phosphomevalonate} \xrightarrow{\text{PMK}} \text{ADP} + \text{pyromevalonate}$$

$$\mathrm{s}_1 + \mathrm{e} \underset{k_{r1}}{\overset{k_{f1}}{\rightleftharpoons}} \mathrm{c}_1 + \mathrm{s}_2 \underset{k_{r2}}{\overset{k_{f2}}{\rightleftharpoons}} \mathrm{c}_2 \xrightarrow{k_{c1}} \mathrm{c}_3 \underset{k_{r3}}{\overset{k_{f3}}{\rightleftharpoons}} \mathrm{c}_4 + \mathrm{p}_1 \underset{k_{r4}}{\overset{k_{f4}}{\rightleftharpoons}} \mathrm{e} + \mathrm{p}_2$$

$$R_5 \begin{cases} \dot{s}_1 = k_{r1a}c_{1a} - k_{f1a}s_1 e + k_{r2b}c_2 - k_{f2b}s_1 c_{1b} \\ \dot{s}_2 = k_{r1b}c_{1b} - k_{f1b}s_2 e + k_{r2a}c_2 - k_{f2a}s_2 c_{1a} \\ \dot{e} = k_{r1a}c_{1a} + k_{r1b}c_{1b} - k_{f1b}s_2 e - k_{f1a}s_1 e + k_{f4a}c_{4a} + k_{f4b}c_{4b} - k_{r4a}p_2 e - k_{r4b}p_1 e \\ \dot{c}_{1a} = k_{f1a}s_1 e - k_{r1a}c_1 a + k_{r2a}c_2 - k_{f2a}s_2 c_{1a} \\ \dot{c}_{1b} = k_{f1b}s_2 e - k_{r1b}c_1 b + k_{r2b}c_2 - k_{f2b}s_1 c_{1b} \\ \dot{c}_2 = k_{f2a}s_2 c_{1a} - k_{r2a}c_2 + k_{f2b}s_1 c_{1b} - k_{r2b}c_2 - k_c c_2 \\ \dot{c}_3 = k_c c_2 + k_{r3a}c_{4a}p_1 - k_{f3a}c_3 + k_{r3b}c_{4b}p_2 - k_{f3b}c_3 \\ \dot{p}_1 = k_{f3a}c_3 - k_{r3a}c_{4a}p_1 + k_{f4b}c_{4b} - k_{r4b}p_1 e \\ \dot{p}_2 = k_{f3b}c_3 - k_{r3b}c_{4b}p_2 + k_{f4a}c_{4a} - k_{r4a}p_2 e \\ \dot{c}_{4a} = k_{f3a}c_3 - k_{r3a}c_{4a}p_1 + k_{f4a}c_{4a} - k_{r4a}p_2 e \\ \dot{c}_{4b} = k_{f3b}c_3 - k_{r3b}c_{4b}p_2 + k_{f4b}c_{4b} - k_{r4b}p_1 e \end{cases}$$

Briggs-Haldane Kinetics:

$$\dot{s} = -\frac{K_{cat}e_0 s}{K_d + s}$$

$$\dot{p} = \frac{K_{cat}e_0 s}{K_d + s}$$

where

$$K_{cat} = k_{c1}$$

$$K_d = \frac{k_{c1} + k_{r1}}{k_{f1}}$$

### 1.6 Reaction 6

PMD proceeds with an ordered sequential reaction mechanism [5]. Ordered sequential mechanism with mevalonate 5-diphosphate as the first substrate to bind to the enzyme [6].

$$\text{diphosphomevalonate} + \text{ATP} \xrightarrow{\text{PMD}} \text{ADP} + \text{phosphate} + \text{isopentenyl diphosphate} + \text{CO}_2$$

$$\mathrm{s_1} + \mathrm{e} \underset{k_{r1}}{\overset{k_{f1}}{\rightleftharpoons}} \mathrm{c_1} + \mathrm{s_2} \underset{k_{r2}}{\overset{k_{f2}}{\rightleftharpoons}} \mathrm{c_2} \xrightarrow{k_{c1}} \mathrm{c_3} \underset{k_{r3}}{\overset{k_{f3}}{\rightleftharpoons}} \mathrm{c_4} + \mathrm{p_1} \underset{k_{r4}}{\overset{k_{f4}}{\rightleftharpoons}} \mathrm{c_5} + \mathrm{p_2} \underset{k_{r5}}{\overset{k_{f5}}{\rightleftharpoons}} \mathrm{c_6} + \mathrm{p_3} \underset{k_{r6}}{\overset{k_{f6}}{\rightleftharpoons}} \mathrm{e} + \mathrm{p_4}$$

$$R_6 \begin{cases} \dot{s}_1 = k_{r1}c_1 - k_{f1}s_1e \\ \dot{e} = k_{r1}c_1 - k_{f1}s_1e + k_{f6}c_6 - k_{r6}p_4e + k_{ri1a}c_{i1a} - k_{fi1a}i_1e + k_{ri1b}c_{i1b} - k_{fi1b}i_2e \\ \dot{c}_1 = k_{f1}s_1e - k_{r1}c_1 - k_{f2}s_2c_1 + k_{r2}c_2 + k_{ri2a}c_{i2a} - k_{fi2a}i_1c_1 + k_{ri2b}c_{i2b} - k_{fi2b}i_2c_1 \\ \dot{s}_2 = k_{r2}c_2 - k_{f2}s_2c_1 \\ \dot{c}_2 = k_{f2}s_2c_1 - k_{r2}c_2 - k_{c1}c_2 \\ \dot{c}_3 = k_{c1}c_2 + k_{r3}p_1c_4 - k_{f3}c_3 \\ \dot{p}_1 = k_{f3}c_3 - k_{r3}p_1c_4 \\ \dot{c}_4 = k_{f3}c_3 - k_{r3}p_1c_4 + k_{r4}p_2c_5 - k_{f4}c_4 \\ \dot{p}_2 = k_{f4}c_4 - k_{r4}p_2c_5 \\ \dot{c}_5 = k_{f4}c_4 - k_{r4}p_2c_5 - k_{f5}c_5 + k_{r5}c_6p_3 \\ \dot{p}_3 = k_{f5}c_4 - k_{r5}p_3c_6 \\ \dot{c}_6 = k_{f5}c_4 - k_{r5}p_3c_6 - k_{f6}c_6 + k_{r6}p_4e \\ \dot{p}_4 = k_{f6}c_6 - k_{r6}p_4e \\ \dot{c}_{i1a} = k_{fi1a}i_1e - k_{ri1a}c_{i1a} - k_{fta}s_1c_{i1a} + k_{rta}c_{i2a} \\ \dot{c}_{i1b} = k_{fi1b}i_2e - k_{ri1b}c_{i1b} - k_{ftb}s_1c_{i1b} + k_{rtb}c_{i2b} \\ \dot{c}_{i2a} = k_{fta}s_1c_{i1a} - k_{rta}c_{i2a} + k_{fi2a}i_1c_1 - k_{ri2a}c_{i2a} \\ \dot{c}_{i2b} = k_{ftb}s_1c_{i1b} - k_{rtb}c_{i2b} + k_{fi2b}i_2c_1 - k_{ri2b}c_{i2b} \\ \dot{i}_1 = k_{ri1a}c_{i1a} - k_{fi1a}i_1e + k_{ri2a}c_{i2a} - k_{fi2a}i_1c_1 \\ \dot{i}_2 = k_{ri1b}c_{i1b} - k_{fi1b}i_2e + k_{ri2b}c_{i2b} - k_{fi2b}i_2c_1 \end{cases}$$

Mixed Inhibition was shown for mevalonate and phosphomevalonate with respect to ATP in the gallus gallus homolog of the enzyme [6].

This is actually competitive inhibition because dual mixed inhibition results in some nasty equations.

$$\dot{s}_1 = -\frac{K_1e_0s}{K_2i_1 + K_3i_2 + K_4s + K_5} \tag{5}$$

$$\dot{p}_1 = \frac{K_1e_0s}{K_2i_1 + K_3i_2 + K_4s + K_5} \tag{6}$$

## 1.7 Reaction 7

IDI mechanism, Irreversible Inhibition Possible. [11]

$$\text{Isopentenyl diphosphate} \xrightarrow{\text{IDI}} \text{Dimethylallyl diphosphate}$$

$$\mathrm{s} + \mathrm{e} \underset{k_{r1}}{\overset{k_{f1}}{\rightleftharpoons}} \mathrm{c} \xrightarrow{\mathrm{k_{c1}}} \mathrm{e} + p$$

$$R_7 \begin{cases} \dot{s} = k_{r1}c - k_{f1}se \\ \dot{e} = k_{r1}c - k_{f1}se + k_{c1}c \\ \dot{c} = k_{f1}se - k_{r1}c - k_{c1}c \\ \dot{p}_1 = k_{c1}c \end{cases}$$

Briggs-Haldane Kinetics:

$$\dot{s} = -\frac{K_{cat}e_0 s}{K_d + s}$$

$$\dot{p} = \frac{K_{cat}e_0 s}{K_d + s}$$

where

$$K_{cat} = k_{c1}$$

$$K_d = \frac{k_{c1} + k_{r1}}{k_{f1}}$$

## 1.8 Reaction 8

$$\text{dimethylallyl diphosphate} + \text{isopentenyl diphosphate} \xrightarrow{\text{GPPS}} \text{diphosphate} + \text{geranyl diphosphate}$$

$$\mathrm{s_1} + \mathrm{e} \underset{k_{r1}}{\overset{k_{f1}}{\rightleftharpoons}} \mathrm{c_1} + \mathrm{s_2} \underset{k_{r2}}{\overset{k_{f2}}{\rightleftharpoons}} \mathrm{c_2} \xrightarrow{k_{c1}} \mathrm{c_3} \underset{k_{r3}}{\overset{k_{f3}}{\rightleftharpoons}} \mathrm{c_4} + \mathrm{p_1} \underset{k_{r4}}{\overset{k_{f4}}{\rightleftharpoons}} \mathrm{e} + \mathrm{p_2}$$

$$R_8 \begin{cases} \dot{s}_1 = k_{r1}c_1 - k_{f1}s_1e \\ \dot{e} = k_{r1}c_1 - k_{f1}s_1e + k_{f4}c_4 - k_{r4}p_2e \\ \dot{c}_1 = k_{f1}s_1e - k_{r1}c_1 - k_{f2}s_2c_1 + k_{r2}c_2 \\ \dot{s}_2 = k_{r2}c_2 - k_{f2}s_2c_1 \\ \dot{c}_2 = k_{f2}s_2c_1 - k_{r2}c_2 - k_{c1}c_2 \\ \dot{c}_3 = k_{c1}c_2 + k_{r3}p_1c_4 - k_{f3}c_3 \\ \dot{c}_4 = k_{f3}c_3 - k_{r3}p_1c_4 + k_{r4}p_2e - k_{f4}c_4 \\ \dot{p}_1 = k_{f3}c_3 - k_{r3}p_1c_4 \\ \dot{p}_2 = k_{f4}c_4 - k_{r4}p_2e \end{cases}$$

Briggs-Haldane Kinetics:

$$\dot{s_1} = -\frac{K_1 e_0 s_1 s_2}{K_2 + K_3 s_1 K_4 s_2 + s_1 s_2}$$

$$\dot{s_2} = -\frac{K_1 e_0 s_1 s_2}{K_2 + K_3 s_1 K_4 s_2 + s_1 s_2}$$

$$\dot{p} = \frac{K_1 e_0 s_1 s_2}{K_2 + K_3 s_1 K_4 s_2 + s_1 s_2}$$

### 1.9 Reaction 9

Limonene Synthase finally makes limonene.

$$\text{geranyl diphosphate} \xrightarrow{\text{LS}} \text{limonene} + \text{diphosphate}$$

$$\mathrm{s} + \mathrm{e} \underset{k_{r1}}{\overset{k_{f1}}{\rightleftharpoons}} \mathrm{c}_1 \xrightarrow{k_{c1}} \mathrm{c}_2 \underset{k_{r2}}{\overset{k_{f2}}{\rightleftharpoons}} \mathrm{c}_3 + \mathrm{p}_1 \underset{k_{r3}}{\overset{k_{f3}}{\rightleftharpoons}} \mathrm{e} + \mathrm{p}_2$$

$$R_9 \begin{cases} \dot{s} = k_{r1} c_1 - k_{f1} s e \\ \dot{e} = k_{r1} c_1 - k_{f1} s e + k_{f3} c_3 - k_{r3} p_2 e \\ \dot{c}_1 = k_{f1} s e - k_{r1} c_1 - k_{c1} c_1 \\ \dot{c}_2 = k_{r2} p_1 c_3 - k_{f2} c_2 + k_{c1} c_1 \\ \dot{c}_3 = k_{f2} c_2 + k_{r3} p_2 e - k_{f3} c_3 \\ \dot{p}_1 = k_{f2} c_3 - k_{r2} p_1 c_3 \\ \dot{p}_2 = k_{f3} c_3 - k_{r3} p_2 e \end{cases}$$

Briggs-Halidane Equations:

$$\dot{s} = -\frac{K_1 e_0 k_{f3} s}{K_1 s + K_2 p_2 + K_3 p_1 s + K_4 p_1 p_2 + K_5 p_1 p_2 + K_6 s + K_7}$$

$$\dot{p}_1 = \frac{e_0 k_{f2} \left(K_1 s + K_2 p_2 - K_3 p_1 s - K_4 p_1 p_2 - K_5 p_1 p_2\right)}{K_1 s + K_2 p_2 + K_3 p_1 s + K_4 p_1 p_2 + K_5 p_1 p_2 + K_6 s + K_7}$$

$$\dot{p}_2 = \frac{K_1 e_0 k_{f3} s}{K_1 s + K_2 p_2 + K_3 p_1 s + K_4 p_1 p_2 + K_5 p_1 p_2 + K_6 s + K_7}$$

$$
\begin{aligned}
K_1 &= k_{c1}k_{f1}k_{f2}\\
K_2 &= k_{c1}k_{f2}k_{r3} + k_{f2}k_{r1}k_{r3}\\
K_3 &= k_{c1}k_{f1}k_{r2}\\
K_4 &= k_{c1}k_{r2}k_{r3}\\
K_5 &= k_{r1}k_{r2}k_{r3}\\
K_6 &= k_{c1}k_{f1}k_{f3} + k_{f1}k_{f2}k_{f3}\\
K_7 &= k_{c1}k_{f2}k_{f3} + k_{f2}k_{f3}k_{r1}
\end{aligned}
$$

## Composite Model

The complete set of reactions and inhibition relationships are given shown in the figure below. The Metabolites are inside of blue rectangles, the enzymes are in green circles. Black arrows indecate forward flow into the next component. Red dashed arrows indicate an inhibition relationship between the two species.

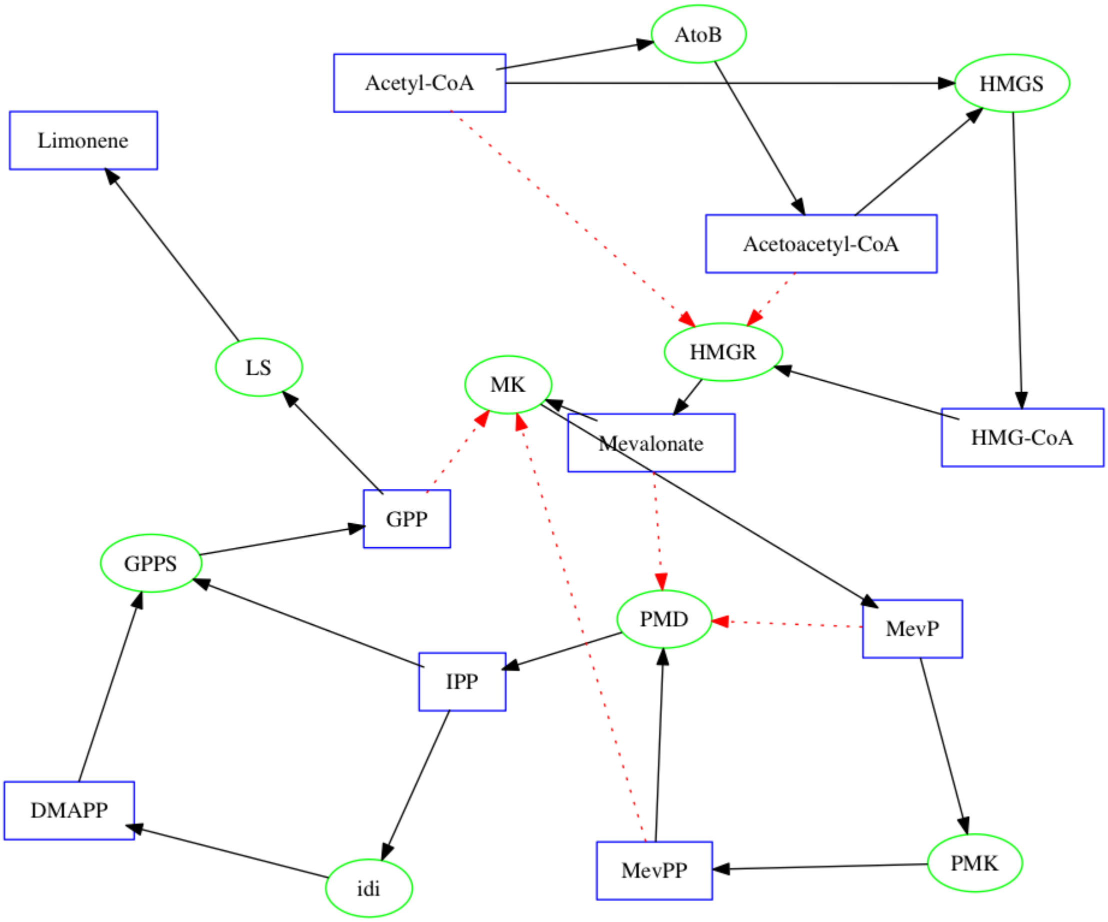

### Reduced Order Michealis Menten Kinetics

Using the relationships derived above from the literature we provide a complete michealis menten description of the system.

$$
\begin{aligned}
\frac{d[A-CoA]}{dt} &= -\frac{K_{1,1}[AtoB][\text{A}-\text{CoA}]}{K_{1,2}+K_{1,3}[A-CoA]} \\
&- \frac{K_{2,1}[HMGS][A-CoA][AA-CoA]k_{s3}}{K_{2,2}[AA-CoA]+K_{2,3}[A-CoA]+K_{2,4}[A-CoA][AA-CoA]} \\
\frac{d[AA-CoA]}{dt} &= \frac{K_{1,1}[AtoB][A-CoA]}{K_{1,2}K_{1,3}[A-CoA]} \\
&- \frac{K_{2,1}[HMGS][A-CoA][AA-CoA]k_{s3}}{K_{2,2}[AA-CoA]+K_{2,3}[A-CoA]+K_{2,4}[A-CoA][AA-CoA]} \\
\frac{d[\text{HMG}-\text{CoA}]}{dt} &= \frac{K_{2,1}[HMGS][A-CoA][AA-CoA]k_{s3}}{K_{2,2}[AA-CoA]+K_{2,3}[A-CoA]+K_{2,4}[A-CoA][AA-CoA]} \\
&- \frac{K_{3,1}[HMGR][HMG-CoA]}{K_{3,2}[A-CoA]+K_{3,3}[AA-CoA]+K_{3,4}[HMG-CoA]+K_{3,5}} \\
\frac{d[\text{Mev}]}{dt} &= \frac{K_{3,1}[HMGR][HMG-CoA]}{K_{3,2}[A-CoA]+K_{3,3}[AA-CoA]+K_{3,4}[HMG-CoA]+K_{3,5}} \\
&- \frac{K_{4,1}[MK][Mev]}{K_{4,2}[GPP]+K_{4,3}[MevP]+K_{4,4}[Mev]+K_{4,5}} \\
\frac{d[\text{MevP}]}{dt} &= \frac{K_{4,1}[MK][Mev]}{K_{4,2}[GPP]+K_{4,3}[MevP]+K_{4,4}[Mev]+K_{4,5}} - \frac{K5,1[PMK][MevP]}{K_{5,1}+[MevP]} \\
\frac{d[\text{MevPP}]}{dt} &= \frac{K5,1[PMK][MevP]}{K_{5,1}+[MevP]} - \frac{K_{6,1}[PMD][MevPP]}{K_{6,2}[MevP]+K_{6,3}[Mev]+K_{6,4}[MevPP]+K_{6,5}} \\
\frac{d[\text{IPP}]}{dt} &= \frac{K_{6,1}[PMD][MevPP]}{K_{6,2}[MevP]+K_{6,3}[Mev]+K_{6,4}[MevPP]+K_{6,5}} - \frac{K_{7,1}[IDI][IPP]}{K_{7,2}+[IPP]} \\
&- \frac{K_{8,1}[GPPS][IPP][DMAPP]}{K_{8,2}+K_{8,3}[IPP]K_{8,4}[DMAPP]+[IPP][DMAPP]} \\
\frac{d[\text{DMAPP}]}{dt} &= \frac{K_{7,1}[IDI][IPP]}{K_{7,2}+[IPP]} - \frac{K_{8,1}[GPPS][IPP][DMAPP]}{K_{8,2}+K_{8,3}[IPP]+K_{8,4}[DMAPP]+[IPP][DMAPP]} \\
\frac{d[\text{GPP}]}{dt} &= \frac{K_{8,1}[GPPS][IPP][DMAPP]}{K_{8,2}+K_{8,3}[IPP]K_{8,4}[DMAPP]+[IPP][DMAPP]} - \frac{K_{9,1}[LS][GPP]}{K_{9,2}+[GPP]} \\
\frac{d[\text{Limonene}]}{dt} &= \frac{K_{9,1}[LS][GPP]}{K_{9,2}+[GPP]}
\end{aligned}
$$

## References

[1] John L Andreassi, Kristina Dabovic, and Thomas S Leyh. Streptococcus pneumoniae isoprenoid biosynthesis is downregulated by diphosphomevalonate: an antimi-

$$\dot{\boldsymbol{m}} = f(\boldsymbol{m}, \boldsymbol{p})$$

| Prediction (output) | Features (input) |
|---|---|
| $\dot{\tilde{m}}_1(t_1)$ | $\tilde{\boldsymbol{m}}(\mathrm{t}_1)$, $\tilde{\boldsymbol{p}}(\mathrm{t}_1)$ |
| ... | ... |
| $\dot{\tilde{m}}_n(t_1)$ | $\tilde{\boldsymbol{m}}(\mathrm{t}_1)$, $\tilde{\boldsymbol{p}}(\mathrm{t}_1)$ |
| ... | ... |
| $\dot{\tilde{m}}_1(t_T)$ | $\tilde{\boldsymbol{m}}(\mathrm{t}_\mathrm{T})$, $\tilde{\boldsymbol{p}}(\mathrm{t}_\mathrm{T})$ |
| ... | ... |
| $\dot{\tilde{m}}_n(t_T)$ | $\tilde{\boldsymbol{m}}(\mathrm{t}_\mathrm{T})$, $\tilde{\boldsymbol{p}}(\mathrm{t}_\mathrm{T})$ |

Figure S1: **Inputs and Outputs to the Machine Learning Model.** The core of this method consists in using machine learning methods to predict the functional relationship between the metabolite derivative and proteomics and metabolomics data, substituting the traditional Michaelis Menten relationship. The machine learning approach involves training a model for each metabolite that is being predicted (Table S1). Each model takes all the measured metabolites and proteins at a particular time $t_i$ as input. The prediction it provides as an output is the derivative of one of the pathway metabolites at the same time instant. The symbols $\tilde{\mathbf{m}}$ and $\tilde{\mathbf{p}}$ denote the experimentally measured metabolomic and proteomics measurements respectively.

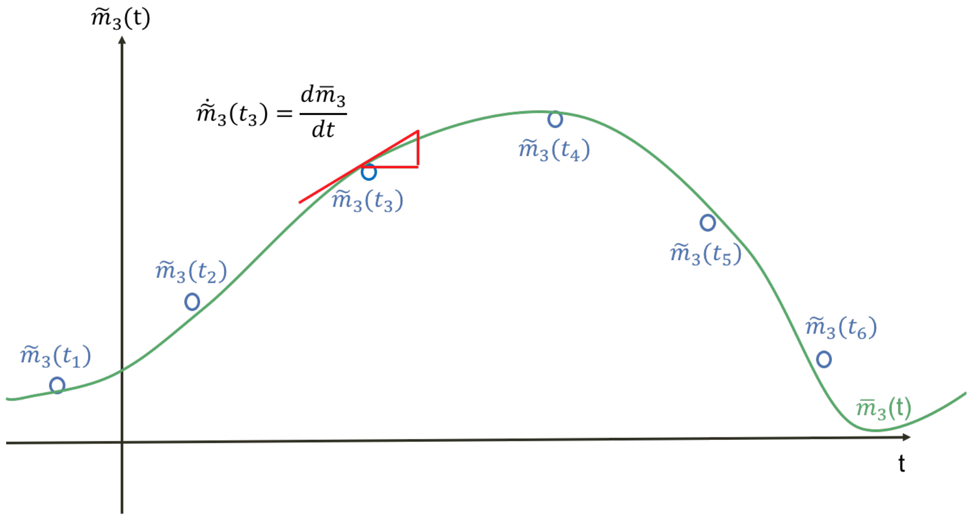


Figure S2: **Computing the Derivative From Metabolomics Training Data.** We start with a set of data points for a particular metabolite. In this case, $\tilde{m}_3$ has been measured at 6 time points. An interpolated and smoothed time series is created from the measurements, $\bar{m}_3(t)$, to reduce the noise of the signal and smooth the resulting derivative. The derivative of the time series is estimated by taking the derivative of the smoothed line at the time point of interest.

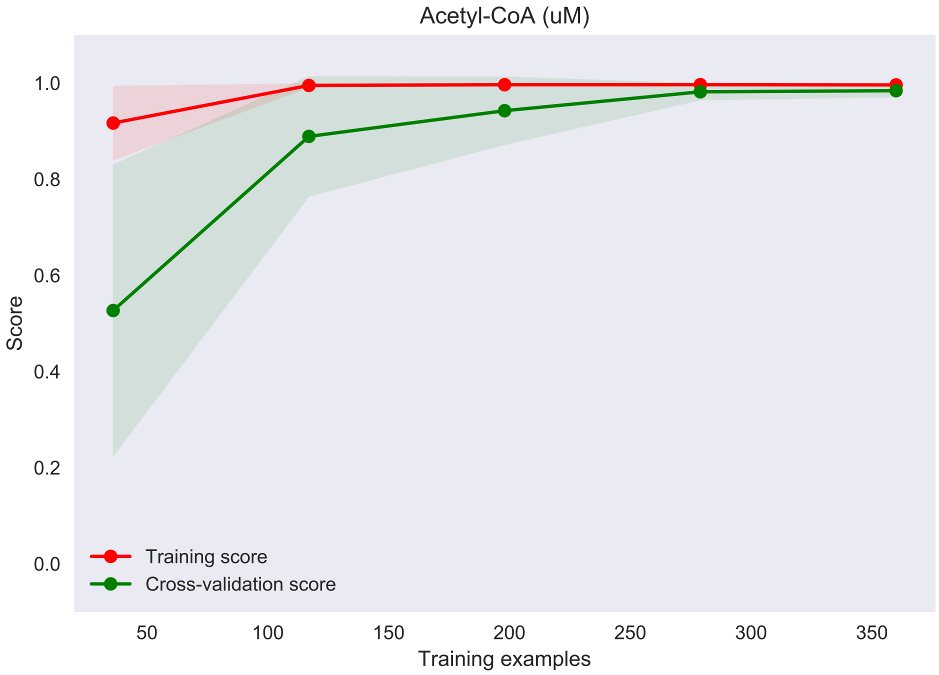


Figure S3: **Cross validation and training scores as a function of training set size.** This is a representative example of how model performance increases with the size of the data set provided. Cross validation techniques train multiple models with a subset of the training data, and then test these model on data not used for training. In this case the training examples involve the time points for which derivatives were calculated from the training data, and proteomics and metabolomics data are available. In the training set each time series contains 7 data points. These are too sparse to formulate accurate models. To overcome this a data augmentation scheme is employed where 7 time points from the original data are expanded into 200 for each strain. This is done by filtering the data and interpolating over the filtered curve. In the plot, two data augmented strains are used where 360 points are used in the training set and 40 points are used in the test set.

| **Pathway** | **Metabolite** | **Machine Learning Model** | Fit Quality (R Value) |
|---|---|---|---|
| Experimental Isopentenol | Acetyl-CoA | Extra Trees Regressor with Polynomial Features | 1.000 |
| | HMG-CoA | Lasso Lars CV → Min Max Scaler → Gradient Boosting Regressor → Decision Tree Regressor | 0.993 |
| | Mevalonate | Extra Trees Regressor | 1.000 |
| | Mev-P | FastICA → LinearSVR → Extra Trees Regressor | 1.000 |
| | IPP/DMAPP | Extra Trees Regressor | 1.000 |
| | Isopentenol | RidgeCV → Extra Trees Regressor | 1.000 |
| Experimental Limonene | Acetyl-CoA | FastICA → PolynomialFeatures → Decision Tree Regressor → FastICS → LassoLarsCV | 0.996 |
| | HMG-CoA | FastICA → One Hot Encoder → Polynomial Features → Max Abs Scaler → K-Neighbors Regressor | 0.944 |
| | Mevalonate | Variance Threshold → RidgeCV → Min Max Scaler → K-Neighbors Regressor | 1.000 |
| | Mev-P | Extra Trees Regressor → Random Forest Regressor → Extra Trees Regressor → Decision Tree Regressor | 0.994 |
| | IPP/DMAPP | Max Abs Scaler → PCA → Max Abs Scaler → Max Abs Scaler → FastICA → Max Abs Scaler → RBFSampler → LassoLarsCV | 0.986 |
| | Limonene | Extra Trees Regressor → Random Forest Regressor | 1.000 |
| Simulated Limonene | Acetyl-CoA | Random Forest Regressor with Polynomial Features | 0.994 |
| | Acetoacetyl-CoA | Random Forest Regressor | 0.997 |
| | HMG-CoA | Extra Trees Regressor | 1.000 |
| | Mevalonate | Extra Trees Regressor | 0.998 |
| | Mev-P | Min-Max Scaler → Robust Scaler → Extra Trees Regressor | 0.997 |
| | Mev-PP | PCA → Extra Trees Regressor | 1.000 |
| | IPP | Extra Trees Regressor | 0.997 |
| | DMAPP | Extra Trees Regressor → LassoLarsCV | 1.000 |
| | GPP | Fast ICA → K-Neighbors Regressor | 1.000 |
| | Limonene | K-Neighbors Regressor | 0.996 |

Table S1: Table containing which machine learning model pipeline was used for each metabolite derivative prediction along with a measure of each models' performance.

crobial target. *Biochemistry*, 43(51):16461–16466, 2004.

[2] Enrique Beytia, J Kevin Dorsey, Jane Marr, WW Cleland, and John W Porter. Purification and mechanism of action of hog liver mevalonic kinase. *Journal of Biological Chemistry*, 245(20):5450–5458, 1970.

[3] David E Garcia and Jay D Keasling. Kinetics of phosphomevalonate kinase from saccharomyces cerevisiae. *PloS one*, 9(1):e87112, 2014.

[4] JC Gray and RGO Kekwick. The inhibition of plant mevalonate kinase preparations by prenyl pyrophosphates. *Biochimica et Biophysica Acta (BBA)-General Subjects*, 279(2):290–296, 1972.

[5] Ana Maria Jabalquinto, Marysol Alvear, and Emilio Cardemil. Physiological aspects and mechanism of action of mevalonate 5-diphosphate decarboxylase. *Comparative Biochemistry and Physiology Part B: Comparative Biochemistry*, 90(4):671–677, 1988.

[6] Ana María Jabalquinto and Emilio Cardemil. Substrate binding order in mevalonate 5-diphosphate decarboxylase from chicken liver. *Biochimica et Biophysica Acta (BBA)-Protein Structure and Molecular Enzymology*, 996(3):257–259, 1989.

[7] Takashi Kawachi and Harry Rudney. Solubilization and purification of $\beta$-hydroxy-$\beta$-methylglutaryl coenzyme a reductase from rat liver. *Biochemistry*, 9(8):1700–1705, 1970.

[8] Daniel Pilloff, Kristina Dabovic, Michael J Romanowski, Jeffrey B Bonanno, Mary Doherty, Stephen K Burley, and Thomas S Leyh. The kinetic mechanism of phosphomevalonate kinase. *Journal of Biological Chemistry*, 278(7):4510–4515, 2003.

[9] Tanja Sgraja, Terry K Smith, and William N Hunter. Structure, substrate recognition and reactivity of leishmania major mevalonate kinase. *BMC structural biology*, 7(1):1, 2007.

[10] Naeem Shafqat, Andrew Turnbull, Johannes Zschocke, Udo Oppermann, and Wyatt W Yue. Crystal structures of human hmg-coa synthase isoforms provide insights into inherited ketogenesis disorders and inhibitor design. *Journal of molecular biology*, 398(4):497–506, 2010.

[11] Johan Wouters, Yamina Oudjama, Victor Stalon, Louis Droogmans, and C Dale Poulter. Crystal structure of the c67a mutant of isopentenyl diphosphate isomerase complexed with a mechanism-based irreversible inhibitor. *Proteins: Structure, Function, and Bioinformatics*, 54(2):216–221, 2004.

| AtoB | HMGS | HMGR | MK | PMK | PMD | Idi | GPPS | LS |
|---|---|---|---|---|---|---|---|---|
| -0.375 | -0.098 | 0.006 | -0.191 | -0.242 | -0.372 | -0.312 | 0.021 | 0.719 |
| -0.018 | 0.426 | 0.504 | -0.274 | 0.446 | -0.259 | -0.422 | -0.078 | -0.193 |

Table S2: **Basis Vectors of Partial Least Squares Regression** The First two components of the Partial Least Squares Regression. These components represent the line that explains the most covariance in the dependent variable of final production.
